# Chirality Sensing of Amino Acid Esters by S-2-Methylbutanamido-Substituted *m*-Phthalic Diamide-Linked Zinc Bisporphyrinate

**DOI:** 10.3390/molecules29153652

**Published:** 2024-08-01

**Authors:** Zhipeng Li, Yue Zhao, Yong Wang, Wen-Hua Zhang, Chuanjiang Hu

**Affiliations:** 1 College of Chemistry, Chemical Engineering, and Materials Science, Soochow University, Suzhou 215123, China; lzp9801@gmail.com (Z.L.); yowang@nju.edu.cn (Y.W.); 2 State Key Laboratory of Coordination Chemistry, School of Chemistry and Chemical Engineering, Nanjing University, Nanjing 210023, China; zhaoyue@nju.edu.cn

**Keywords:** bisporphyrin, chirality sensing, amino acid ester, amido, circular dichroism

## Abstract

To understand the role of an additional coordination site in the linker in chirality sensing, we designed and synthesized an S-2-methylbutanamido-substituted *m*-phthalic diamide-linked zinc bisporphyrinate, [Zn_2_(S-MAABis)] and investigated its ability to sense the chirality of amino acid esters. The ^1^H NMR spectra and the crystal structure showed that the amido oxygen adjacent to the chiral carbon was coordinated with zinc. NMR and UV–vis titration showed that the binding of [Zn_2_(S-MAABis)] to amino acid esters occurred via two equilibria, forming 1:1 and 1:2 host–guest complexes. The CD spectra suggested that [Zn_2_(S-MAABis)] can effectively recognize the absolute configuration of amino acid esters. The sign of the CD spectra remained unchanged during the titration, indicating that the corresponding 1:1 and 1:2 host–guest complexes had the same chirality. This is different from previously studied amino-substituted *m*-phthalic diamide-linked zinc bisporphyrinate [Zn_2_(AmBis)], which showed chirality inversion during titration. Theoretical calculations indicated that the additional coordination sites (amido or amino) in the 1:1 host–guest complexes played different roles, leading to differences in chirality. Our studies suggest that the introduction of a coordination site can influence the chirality transfer process, but the results of chirality transfers are dependent on the specific binding modes.

## 1. Introduction

Due to the strong coupling interactions between two chromophores, bisporphyrins have been widely used in chirality sensing, chirality recognition, etc., in recent years [1,2,3,4,5]. In the reported bisporphyrins, there are various linkers. For example, Borovkov and Inoue synthesized an ethane-linked bisporphyrin [6], which can sense the absolute configurations of diamines, diols, monoamines, etc. Nina and coworkers developed a bisporphyrin with a pentanediol spacer [4,7]. Its metalloporphyrins can sense the chirality of diamines, amino alcohols, amino acids, etc. Hayashi et al. reported an ester-linked bisporphyrin [8], and the flexible linker made zinc bisporphyrinate suitable for sensing monoalcohols. Some chiral bisporhyrins have also been developed, and they have shown enantioselectivity toward chiral guests. For example, Crossley et al. synthesized bismetalloporphyrin analogs of Troger’s base that showed enantioselective binding of histidine esters and lysine esters [9]. Ema and Sakai used a relatively flexible chiral bisporphyrin tweezer with an isophthalic linker, which functioned as a sensitive chiral shift reagent for chiral diamine derivatives [10]. Jiang and Bian developed 1,1′-binaphthalene-bridged bisporphyrins [11,12] that showed chiral discrimination abilities toward 1,2-diamines.

**Figure 1:**
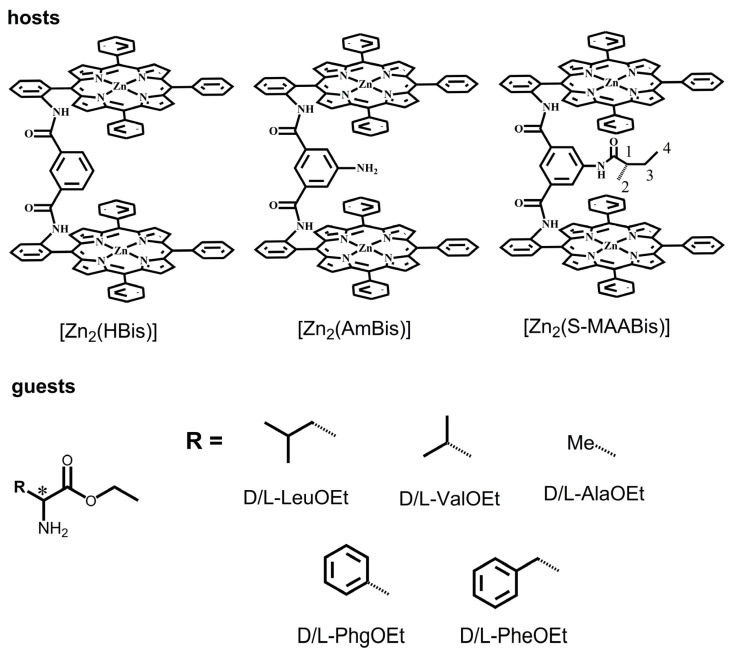
Structural formulas of [Zn_2_(HBis)], [Zn_2_(AmBis)], and [Zn_2_(S-MAABis)] and amino acid esters used in this work.

Our group has been working on amide-linked bisporphyrins in recent years [13,14,15,16,17,18,19,20,21]. Our previous studies suggested that the linkers in *m*-phthalic diamide-linked zinc bisporphyrinates play crucial roles in the process of chirality sensing and recognition. The CD spectral changes for [Zn_2_(AmBis)] during the titration with amino acid esters are significantly different from those of [Zn_2_(HBis)] (Figure 1) [13,20]. Unlike [Zn_2_(HBis)], the linker in [Zn_2_(AmBis)] has an additional coordination site. The CD spectra of [Zn_2_(AmBis)] upon the addition of amino acid esters show chirality inversion, while those of [Zn_2_(HBis)] do not. Our studies suggest that, in the 1:1 host–guest complex [Zn_2_(AmBis)(l-LeuOEt)], the amino nitrogen in the linker is coordinated with zinc, which leads to a different chirality from that of the 1:2 host–guest complex [Zn_2_(AmBis)(l-LeuOEt)_2_]. This results in a stoichiometry-controlled chirality inversion.

The coordination site in the linker is the key factor in controlling the above chirality transfer process. Will the additional coordination site always lead to chirality inversion? To answer this question, we designed a new bisporphyrin, as shown in Figure 1, [Zn_2_(S-MAABis)], which also contains a potential coordination site. In addition, the substituent in the linker is chiral, and we hope to develop the chiral recognition ability of the porphyrin host for enantiomers. We performed detailed investigations by CD, UV–vis spectroscopy, NMR, and DFT calculations.

## 2. Results and Discussion

### 2.1. Characterization of [Zn_2_(S-MAABis)]

#### 2.1.1. Analysis of NMR Spectra

**Figure 2:**
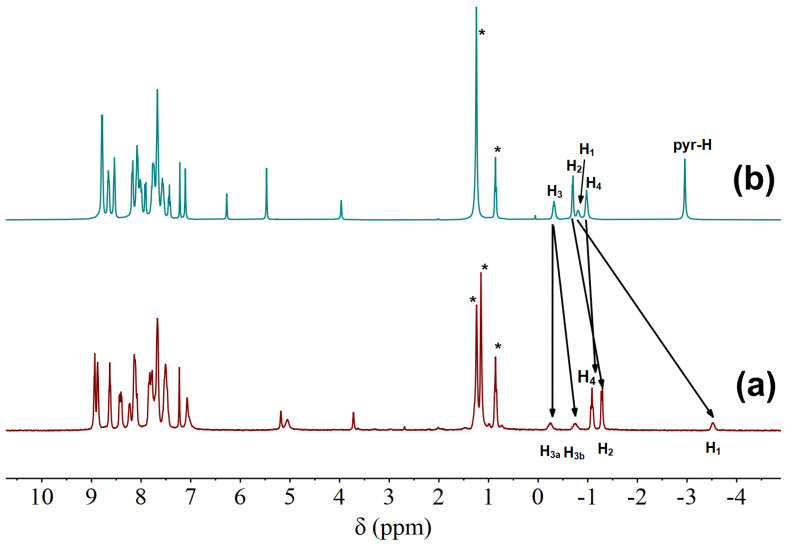
^1^H NMR spectra of (**a**) [Zn_2_(S-MAABis)] (6.1 × 10^−3^ M) and (**b**) H_4_(S-MAABis) (6.1 × 10^−3^ M) in CDCl_3_ at 298 K. * Impurities, such as water and petroleum ether. The arrows represent the changes in chemical shifts of the corresponding hydrogens.

The NMR spectra of H_4_(S-MAABis) and [Zn_2_(S-MAABis)] were measured and are shown in Figure 2. The assignments were based on the ^1^H NMR and ^1^H-^1^H COSY spectra (see Supplementary Materials). For H_4_(S-MAABis), the peak at −3.0 ppm is a typical resonance signal for pyrrole hydrogen (pyr-H) within the porphyrin ring [22]. For the remaining signals, we were concerned about the signals of protons in the substituent in the linker, as shown in Figure 2. H_1_, H_2_, H_3_, and H_4_ were assigned to the signals at −0.82 ppm, −0.72 ppm, −0.35 ppm, and −0.99 ppm, respectively.

For [Zn_2_(S-MAABis)], the disappearance of the signal at −3.0 ppm indicated complete metalation. As shown in Figure 2, there were five different peaks in the range of 0 to −3.7 ppm, and their integration ratio was 1:1:3:3:1. The signals at −0.31 ppm, −0.80 ppm, −1.14, −1.34 ppm, and −3.58 ppm were assigned to H_3a_, H_3b_, H_4_, H_2_, and H_1_, respectively. The splitting of the H_3_ signals indicates that they were influenced by the chiral carbon, and the chemical environments of the two hydrogens were no longer equivalent.

Compared with those of 2-methylbutyric acid (Figure S3), these resonance signals all shifted upfield, which suggests that these hydrogens are located above the porphyrin plane. Due to the influence of the porphyrin ring current, the corresponding NMR signals shifted upfield [22]. As shown in Figure 2, upon metalation, the resonance signal of H_1_ shifted further upfield by 2.76 ppm. This significant upfield shift suggests that H_1_ in [Zn_2_(S-MAABis)] is much closer to the porphyrin plane than that in H_4_(S-MAABis). It is highly likely that the amido oxygen of the substituent in [Zn_2_(S-MAABis)] coordinates with zinc, which pulls those hydrogens near the carbonyl oxygen closer to the porphyrin plane. The coordination interactions were further confirmed by the crystal structure.

#### 2.1.2. Crystal Structure of [Zn_4_(MAABis)_2_(H_2_O)]

We attempted to cultivate single crystals of optically pure [Zn_2_(S-MAABis)], but did not obtain any suitable single crystals. Alternatively, we prepared racemic [Zn_2_(MAABis)] and obtained single crystals suitable for X-ray diffraction by diffusing n-hexane into a CH_2_Cl_2_ solution of [Zn_2_(MAABis)]. Although the quality of the single-crystal data was not very good, these data can still provide useful information on the substituent in the linker.

**Figure 3:**
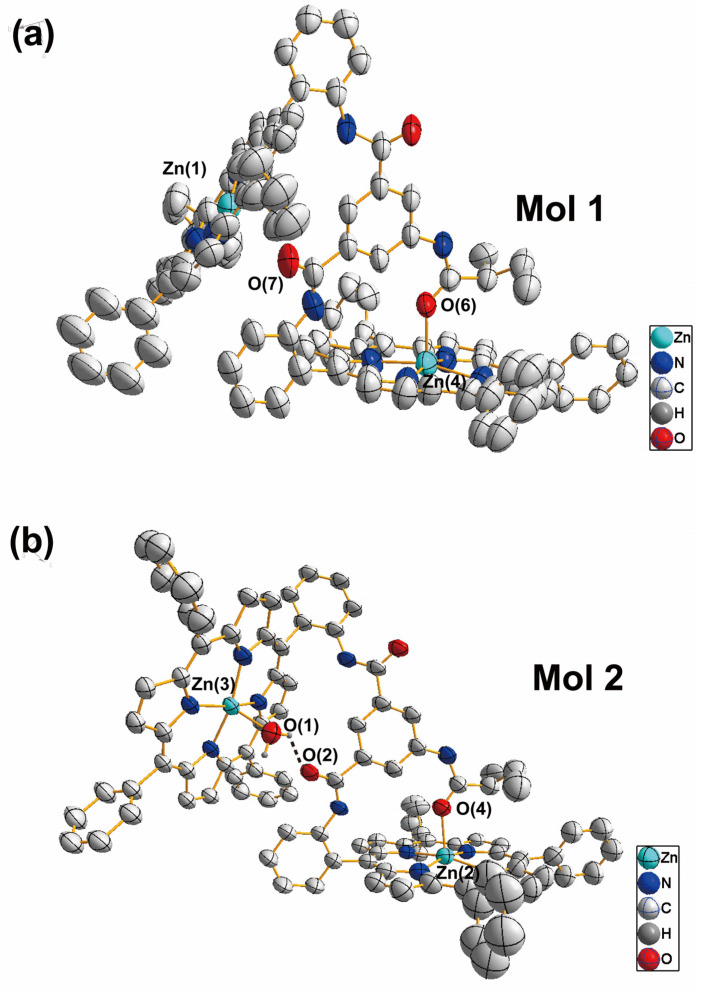
The crystal structure of [Zn_4_(MAABis)_2_(H_2_O)], with an anisotropic displacement ellipsoid at a probability level of 30%. (**a**) Mol **1.**; (**b**) Mol **2**. Zn(4)−O(6) = 2.165 (6) Å, Zn(2)−O(4) = 2.215 (6) Å, Zn(3)−O(1) = 2.155 (6) Å, O(1)···O(2) = 2.765 Å.

The [Zn_4_(MAABis)_2_(H_2_O)] compound was crystallized in a monoclinic crystal system with a *P*2_1_/*n* space group. Each asymmetric unit contained two zinc bisporphyrinate molecules (Mol 1 and Mol 2) (Figure 3). In Mol 1, Zn(1) was four-coordinate, while Zn(4) was five-coordinate. For Zn(4), in addition to the four pyrrole nitrogen atoms, the fifth coordinating atom was the amido oxygen O(6) in the linker. In Mol 2, Zn(2) had a coordination environment similar to that of Zn(4) and was also axially ligated by the carbonyl oxygen O(4) in the linker. However, unlike Mol 1, the other Zn(3) in this bisporphyrin was also five-coordinate, with water O(1) as the axial ligand. Water also formed hydrogen bonds with the amido oxygen O(2) in the linker, with a corresponding O(1)···O(2) distance of 2.765 Å. In addition, there were double hydrogen bonds between Mol 1 and Mol 2: one was between O(5) and N(20), and the other was between O(3) and N(17) (Figure S4).

### 2.2. Chirality Sensing and Recognition of Amino Acid Esters by [Zn_2_(S-MAABis)]

The above studies on [Zn_2_(S-MAABis)] indicate that there is a coordination interaction between the amido oxygen and zinc within the zinc bisporphyrinate. What is the impact of this coordination interaction on its chirality sensing and recognition ability? We further investigated its interactions with amino acid esters.

#### 2.2.1. CD Spectra upon Complexation of Zinc Bisporphyrinate with Amino Acid Esters

**Figure 4:**
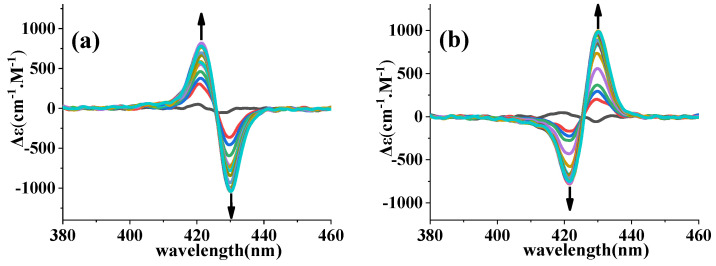
CD spectra of a mixture of [Zn_2_(S-MAABis)] (1.0 × 10^−6^ M) and 0–1000 equivalents of guest (**a**) l-LeuOEt and (**b**) d-LeuOEt in a mixed solvent (n-hexane: dichloromethane = 4:1) at 298 K.

We used [Zn_2_(S-MAABis)] as the host and amino acid esters as the guests. The following analysis used leucine ethyl ester as an example. As shown in Figure 4, before the addition of leucine ethyl ester, the CD spectrum showed a very weak signal for this chiral zinc bisporphyrinate. When l-LeuOEt was added dropwise, the CD spectra showed negative exciton chirality: the signal at a longer wavelength (430 nm) was negative, while the signal at a shorter wavelength (421 nm) was positive. For d-LeuOEt, the CD spectra showed positive exciton chirality: the signal at 430 nm was positive, while the signal at 421 nm was negative. The CD spectral data are summarized in Table 1. The CD spectra indicated that [Zn_2_(S-MAABis)] has the ability to recognize the absolute configuration of amino acid esters and can serve as a chirality sensor for amino acid esters. We also noticed that there was no change in the sign of the CD spectra during titration, only a change in intensity. This is different from the titration of [Zn_2_(AmBis)], which showed positive exciton chirality at low concentrations of l-LeuOEt and negative exciton chirality at high concentrations of l-LeuOEt.

For all the amino acid esters, the CD spectra were similar. When there was a large excess of guests, the CD intensity reached saturation. The corresponding CD spectra of the mixture of [Zn_2_(S-MAABis)] and amino acid esters are shown in Figures S5–S9.

#### 2.2.2. NMR Studies

**Figure 5:**
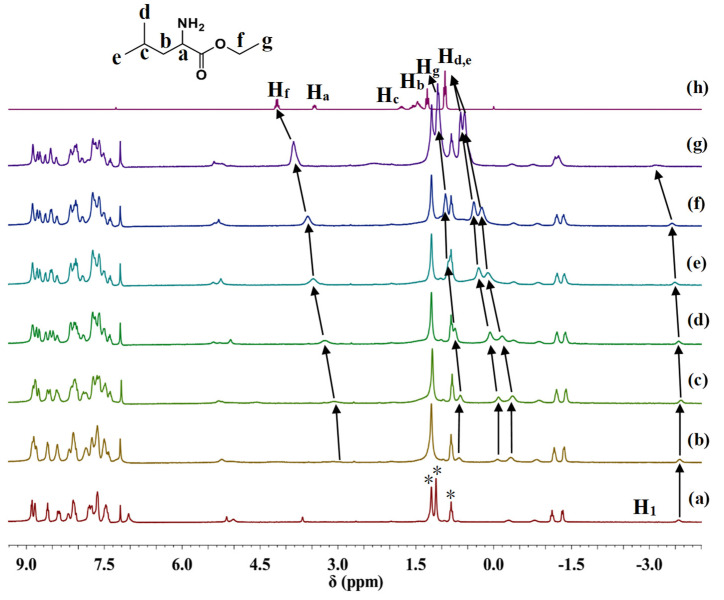
^1^H NMR spectra of [Zn_2_(S-MAABis)] in CDCl_3_ (6.0 × 0^−3^ M) with l-LeuOEt at 298 K. The equivalents of l-LeuOEt are (**a**) 0 eq., (**b**) 0.4 eq., (**c**) 0.8 eq., (**d**) 1.0 eq., (**e**) 1.8 eq., (**f**) 2.2 eq., (**g**) 3.2 eq., and (**h**) l-LeuOEt. * Impurities, such as water and petroleum ether. The arrows represent the changes in chemical shifts of the corresponding hydrogens.

To understand the chiral transfer process, we performed ^1^H NMR measurements on a solution of [Zn_2_(S-MAABis)] after the addition of l-leucine ethyl ester. The results are shown in Figure 5. The assignments of some signals of leucine ethyl ester were based on the titration spectra and comparison with the titration NMR spectra of other amide-linked zinc bisporphrinates [20].

When the l-LeuOEt concentration was 0.4 equivalents, the resonance signals at −0.29, −0.03, and 0.70 ppm were assigned to H_d_, H_e_, and H_g_, respectively, and the signals at 3.0 ppm were assigned to H_f_. Other signals were too broad to be observed. Obviously, the NMR resonance signals of amino acid esters significantly shifted upfield compared with those of uncoordinated amino acid esters, further indicating the coordination between amino acid esters and zinc. The coordination interactions caused amino acid esters to be located over the porphyrin plane, and the ring current effect of porphyrin caused its signal to shift upfield.

In addition, we were interested in the changes in the resonance signals of chiral substituents during the titration. As shown in Figure 5, the resonance signal of H_1_ before titration was at −3.6 ppm. When the amount of l-LeuOEt added was less than 1.0 equivalents, the signal basically did not shift, indicating that, at low concentrations of amino acid esters, the coordination with zinc porphyrinate does not affect the hydrogen of the chiral carbon in the linker, which suggests that amino acid esters bind to the four-coordinate zinc. When the concentration of L-leuOEt gradually increased and exceeded 1.0 equivalents, the H_1_ signal shifted significantly, indicating that amino acid esters began to replace the coordination of amido oxygen in the linker. The above results indicate that the coordination process between amino acid esters and zinc bisporphyrinate can be divided into two steps: at low concentrations of amino acid esters, they combine with the four-coordinate zinc porphyrinate to form a 1:1 host–guest complex [Zn_2_(S-MAABis)L]; at high concentrations of amino acid esters, the amino acid esters replace the coordination of the linker, forming a 1:2 host–guest complex [Zn_2_(S-MAABis)L_2_]. (L represents the amino acid ester ligand.) Zinc is generally five-coordinate in these zinc porphyrinate systems [4,23,24,25,26], so in these 1:1 host–guest complexes and 1:2 host–guest complexes, the zinc atoms were all five-coordinate.

(1) $$[{\mathrm{Zn}}_{2}(S-\mathrm{MAABis})]+L\overset{K_{1}}{⇌}\left[{\mathrm{Zn}}_{2}(S-\mathrm{MAABis})L\right]$$

(2) $$[{\mathrm{Zn}}_{2}(S-\mathrm{MAABis})L]+L\overset{K_{2}}{⇌}\left[{\mathrm{Zn}}_{2}(S-\mathrm{MAABis})L2\right]$$

We were also concerned about the enantioselectivity of this chiral zinc bisporphyrinate toward amino acid esters via NMR. Therefore, we also performed ^1^H NMR measurements on a solution of [Zn_2_(S-MAABis)] after the addition of d-leucine ethyl ester. As shown in Figure S14, the spectra were similar to that for l-leucine ethyl ester. These NMR results suggested that d-/l-LeuOEt could hardly be distinguished by [Zn_2_(S-MAABis)].

#### 2.2.3. UV–Vis Spectrophotometric Titration

**Figure 6:**
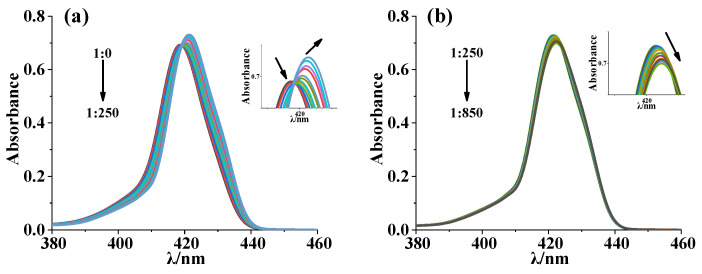
UV–visible titration spectra of a mixture of [Zn_2_(S-MAABis)] (1.0 × 10^−6^ M) and l-LeuOEt in a mixed solvent (n-hexane:dichloromethane = 4:1) at 298 K, with host–guest ratios ranging from (**a**) 1:0 to 1:250 and (**b**) 1:250 to 1:850. The arrows represent the changes in maxium absorptions.

We further determined the corresponding equilibrium constants by a UV–vis spectrophotometric titration method. We titrated the [Zn_2_(S-MAABis)] solution with amino acid ethyl ester and measured the corresponding UV–vis spectra, as shown in Figure 6 and Figures S15–S23. Taking l-LeuOEt as an example, before the addition of l-LeuOEt, [Zn_2_(S-MAABis)] exhibited a strong absorption peak at 419 nm in the Soret band. As l-LeuOEt was added dropwise, the overall change can be divided into two steps: as the amount of l-leucine ethyl ester increased from 0 to 250 eq, the peak intensity at 419 nm gradually decreased, and a new band appeared at 421 nm and gradually strengthened. When the amount of l-leucine ethyl ester increased from 250 eq, the peak intensity at 421 nm slightly decreased and gradually shifted to 423 nm. For the other four amino acid ethyl esters, the spectral changes during titration were similar. The two-step changes in the UV–visible spectra mentioned above were consistent with the two equilibria shown in Equations (1) and (2) and were also consistent with the NMR results. As described in Equilibrium (1) and Equilibrium (2), 1:1 and 1:2 host–guest complexes can be formed between zinc porphyrinate and amino acid esters. When the concentrations of amino acid esters are low, the 1:1 host–guest complex [Zn_2_(S-MAABis)L] is the dominant CD-active species. At higher guest concentrations, the 1:2 host–guest complex [Zn_2_(S-MAABis)L_2_] is dominant. The CD spectra did not show any changes in sign during titration, indicating that the sign of the CD signals of the 1:1 host–guest complex [Zn_2_(S-MABis)L] and the 1:2 host–guest complex [Zn_2_(S-MAABis)L_2_] were the same. This result is different from the titration result of [Zn_2_(AmBis)], which showed chirality inversion during the titration of amino acid esters [13].

For the two abovementioned equilibria, their binding constants were calculated with the SQUAD program [27]. The results are listed in Table 2. The equilibrium constant K_1_ ranged from 2.4 × 10^4^ to 1.8 × 10^5^, and K_2_ ranged from 3.1 × 10^2^ to 1.2 × 10^3^. K_1_ was much larger than K_2_. These results are similar to those observed in other amide-linked bisporphyrin systems [14].

During the titration process, [Zn_2_(S-MAABis)] was converted to [Zn_2_(S-MAABis)(l-LeuOEt)] and [Zn_2_(S-MAABis)(l-LeuOEt)_2_]. However, the corresponding equilibrium constants K_1_ and K_2_ were not very large. Since the host concentration was 1.0 × 10^−6^ mol/L for UV–vis measurements, when the guest concentration was around 250 eq., the calculations suggested that [Zn_2_(S-MAABis)] was not completely converted to a 1:1 complex or a 1:2 complex according to the equilibrium constants in Table 2. At 250 equivalents of l-LeuOEt, the calculated concentration ratio of [Zn_2_(S-MAABis)(l-LeuOEt)_2_] to [Zn_2_(S-MAABis)(l-LeuOEt)] was 0.3, indicating that [Zn_2_(S-MAABis)(l-LeuOEt)] was more dominant than [Zn_2_(S-MAABis)(l-LeuOEt)_2_] in solution. As the guest concentrations continued to increase, the amount of the 1:2 complex increased and the ratio of [Zn_2_(S-MAABis)(l-LeuOEt)_2_] to [Zn_2_(S-MAABis)(l-LeuOEt)] also increased. Due to the different UV–visible absorption of 1:2 and 1:1 complexes, this led to changes in the UV–vis spectra during titration.

The reagent [Zn_2_(S-MAABis)] can be recovered by removing the solvent, washing with methanol, and recrystallizing. Due to the use of only a small amount of [Zn_2_(S-MAABis)] in mM for each CD or UV–vis measurement, we usually recovered it after multiple measurements.

For this chiral zinc bisporphyrinate, we also calculated the corresponding enantioselectivity coefficient (α). The enantioselectivity coefficient reflects the ability of chiral hosts to selectively recognize the enantiomers of guests. As shown in Table 2, the enantioselectivity coefficients were all around 1, indicating a poor enantioselectivity of [Zn_2_(S-MAABis)] towards amino acid esters. This may be because its chiral substituent is far from the amino acid ester. Theoretical calculations also verified this point (via infra).

#### 2.2.4. Computational Studies

**Figure 7:**
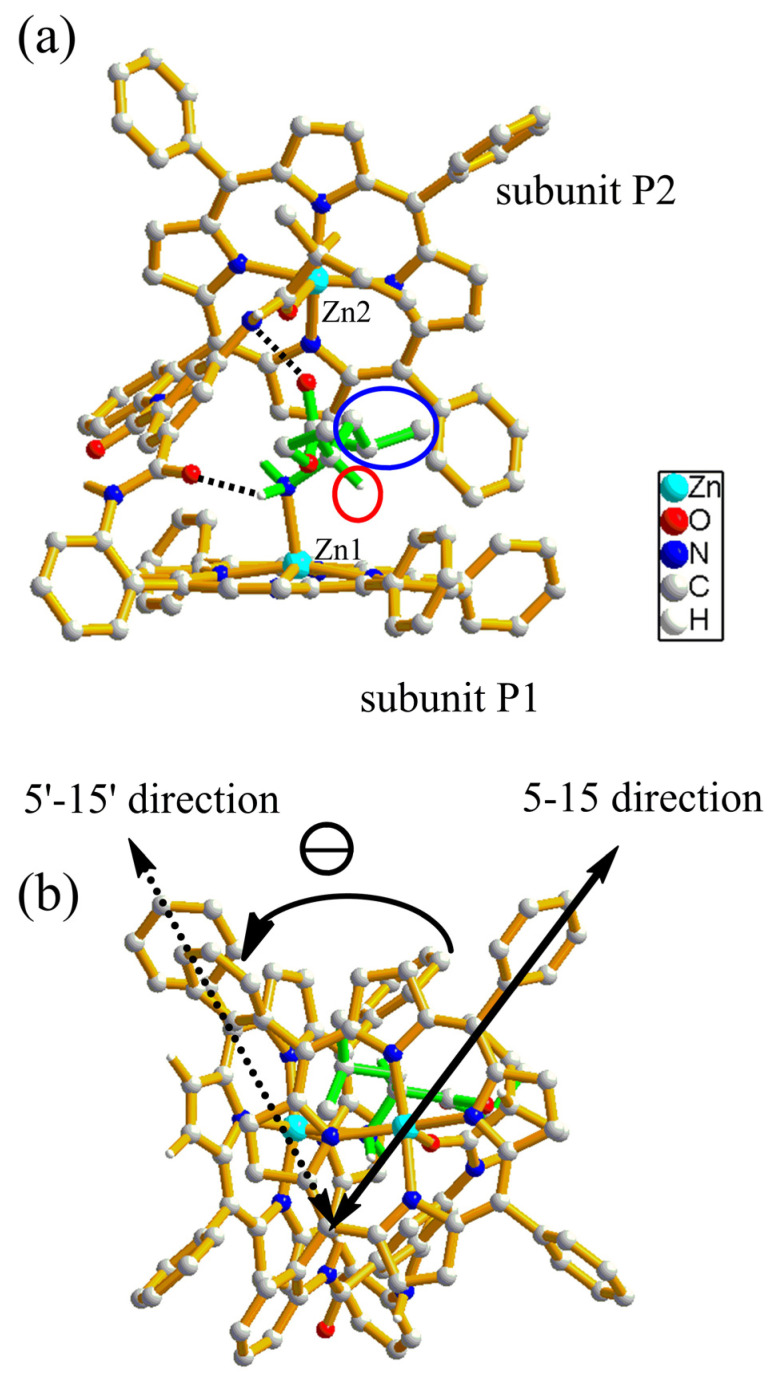
(**a**) Optimized structure of [Zn_2_(S-MAABis)(l-LeuOEt)]. The hydrogen of the chiral carbon atom is marked by a red circle, and the isobutyl group is marked by a blue circle. Hydrogen bonds are labeled with dashed lines. (**b**) The anticlockwise twist angle formed between the transition dipole moments in [Zn_2_(S-MAABis)(l-LeuOEt)]. l-LeuOEt molecules are highlighted in green.

**Figure 8:**
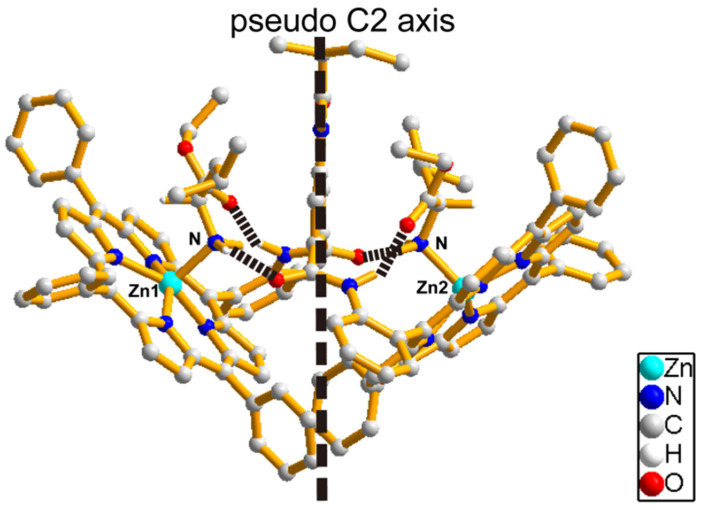
The optimized structure of [Zn_2_(S-MAABis)(L-LeuOEt)_2_]. The dashed lines show hydrogen bonds.

The above studies indicate that [Zn_2_(S-MAABis)] interacts with amino acid esters to form 1:1 and 1:2 host–guest complexes through two equilibria, while the sign of the CD spectra remains unchanged during titration. To understand the CD spectra, DFT calculations were performed for the 1:1 and 1:2 host–guest complexes with [Zn_2_(S-MAABis)] as the host and l-LeuOEt as the guest. The optimized structures were obtained and are displayed in Figure 7 and Figure 8.

With respect to the optimized structure of the 1:1 host–guest complex [Zn_2_(S-MAABis)(l-LeuOEt)], we labeled the zinc porphyrinate subunit coordinated with the amino acid ethyl ester as P1 and the other as P2. In addition to coordinating with the four nitrogen atoms in the porphyrin, the zinc in P1 also coordinated with the amino nitrogen atom of the amino acid ester, and the zinc in P2 also coordinated with the amido oxygen in the linker. Therefore, both zinc atoms were five-coordinate.

In addition to the coordination interactions, this structure exhibited two hydrogen bonds, as shown in Figure 7: one was formed between the amino hydrogen of the ligand and the amido oxygen adjacent to subunit P1, and the other was formed between the carbonyl oxygen of the ester group in the ligand and the amido hydrogen adjacent to the chiral carbon in the linker. The corresponding distances of NH···O (hydrogen-to-oxygen) were 2.30 and 2.65 Å, respectively. As shown in Figure 7, it was also clear that the S-2-methylbutanamido substituent was far from the amino acid ester, which could be the reason why this chiral zinc bisporphyrinate has a poor enantioselectivity toward amino acid esters (via supra).

What is the structural feature leading to the corresponding CD signals? For leucine ethyl ester, the chiral carbon is bonded with four different functional groups: NH_2_, an ethyl ester group, hydrogen, and an isobutyl group. Due to coordination and hydrogen bonding, the positions of the NH_2_ and ethyl ester groups are relatively fixed. In the optimized structure, the smaller hydrogen faces the porphyrin plane, while the larger isobutyl group moves away from the porphyrin plane. This configuration is beneficial for reducing the steric repulsion between amino acid ester substituents and porphyrin planes; if replaced with d-type leucine ethyl ester, the larger isobutyl group will face the porphyrin plane, resulting in greater steric repulsion with the porphyrin plane. Therefore, in the optimized structure shown in Figure 7, hydrogen bonding, coordination, and steric repulsion all contribute to the most energetically favorable conformation. The corresponding torsion angle of 15-5-5′-15′ is −63° in the optimized structure. According to the exciton coupling method, a negative torsion angle corresponds to negative chirality, which is consistent with the experimental results.

Why does CD inversion occur for [Zn_2_(AmBis)] during the titration of amino acid esters, but not for [Zn_2_(S-MAABis)], even though both of these compounds contain an additional coordination site in the linker? The above studies suggest that their 1:1 host–guest complexes are responsible for the different CD spectra. What is the difference in structure between their 1:1 host–guest complexes? We compared the optimized structure of [Zn_2_(S-MAABis)(l-LeuOEt)] with that of [Zn_2_(AmBis)(l-LeuOEt)] [13]. Obviously, the roles of the coordination groups in these two structures are different. The amido groups in [Zn_2_(S-MAABis)(l-LeuOEt)] participate not only in coordination, but also in hydrogen bonding. The amino groups in [Zn_2_(AmBis)(l-LeuOEt)] only participate in coordination, which leads to different patterns of coordination and hydrogen bonding in the two structures, and the corresponding conformations of the bisporphyrins are also completely different. The former has a negative 15-5-5′-15′ torsion angle, while the latter has a positive value. According to the exciton coupling method, the two complexes have different chiralities, resulting in different CD signals.

For the 1:2 host–guest complex, the optimized structure of [Zn_2_(S-MAABis)(l-LeuOEt)_2_] is shown in Figure 8, where two zinc atoms are both five-coordinate with four pyrrole nitrogen atoms and one amino nitrogen atom. Similar to the previously studied 1:2 host–guest complex [Zn_2_(AmBis)(l-LeuOEt)_2_], there were also four hydrogen bonds in [Zn_2_(S-MAABis)(l-LeuOEt)_2_]: two hydrogen bonds were formed between the ligated NH_2_ of the amino acid esters and the carbonyl oxygen in the linker, and another two were formed between the carbonyl oxygen of the amino acid esters and the amido NH in the linker. The corresponding NH···O distances were 2.23 Å, 1.86 Å, 1.94 Å, and 2.11 Å.

**Figure 9:**
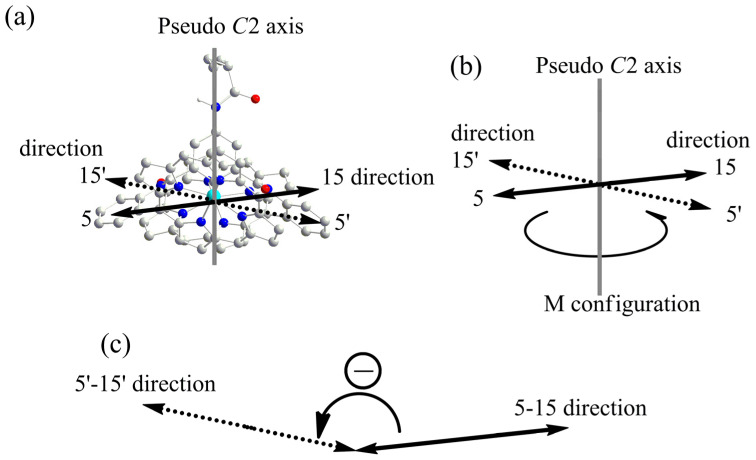
Illustration of the chirality arrangement in the optimized structure of [Zn_2_(S-MAABis)(l-LeuOEt)_2_]. (**a**) The transition dipole moments in the 5–15 and 5′–15′ directions. (**b**) Illustration of the M configuration in the structure. (**c**) The anticlockwise twist with a negative 15–5–5′–15′ torsional angle.

As shown in Figure 8 and Figure 9, the entire structure exhibited pseudo *C*2 symmetry. Due to the spatial arrangement of the porphyrin units, the 1:2 host–guest complex exhibited left-handed helical chirality (M configuration). In the optimized structure of [Zn_2_(S-MAABis)(l-LeuOEt)_2_], the transition dipole moments of the two porphyrin units formed an anticlockwise twist with a value of -163°. According to the exciton chirality method, a negative twist angle should lead to negative exciton chirality. This is also consistent with the experimental results.

**Figure 10:**
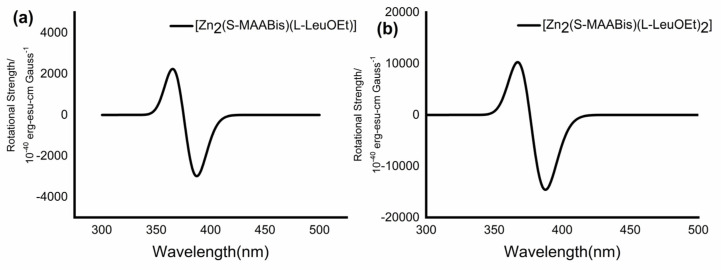
(**a**) Calculated CD spectra for a) [Zn_2_(S-MAABis)(l-LeuOEt)] and (**b**) [Zn_2_(SMAABis)(l-LeuOEt)_2_] at the WB97XD/6−31G** level.

The TDDFT calculations were also performed on the optimized structures of [Zn_2_(S-MAABis)(l-LeuOEt)] and [Zn_2_(S-MAABis)(l-LeuOEt)_2_]. The simulated electronic CD spectra are shown in Figure 10, and are also in accordance with the experimental results.

## 3. Materials and Methods

### 3.1. General

Dichloromethane was distilled after refluxing with calcium hydride. Freshly distilled amino acid esters were prepared according to previously reported methods [28]. H_4_(AmBis) was synthesized according to our previous report [13]. Other chemicals and reagents were commercially available and used without further purification.

The FT-IR spectra were measured on a Varian 1000 FT-IR spectrometer (Varian, Inc., Palo Alto, CA, USA) as KBr disks (400–4000 cm^−1^). Elemental analyses for C, H, and N were carried out on a Carlo-Erba CHNO-S microanalyzer (Carlo Erba, Waltham, MA, USA). The UV–vis spectra were obtained using a Shimadzu UV-3150 spectrometer (Shimadzu Corporation, Kyoto, Japan), the instrument used for ^1^H NMR spectroscopy was a Bruker AVANCE 400 MHz, and the CD spectrum was obtained using a PMT Model J-815 spectrometer (JASCO, Tokyo, Japan). The scanning conditions were as follows: wavelength step = 1.00 nm, bandwidth = 2 nm, response time = 0.1 s, and scanning speed = 200 nm/min.

In all the UV–vis or CD measurements, the solvents were anhydrous. The UV–vis and CD titration experiments were carried out as follows. Different amounts of an optically active amino acid ester solution were added to the [Zn_2_(S-MAABis)] solution in CH_2_Cl_2_ at 298 K. Then, UV–vis or CD spectra were recorded. CD spectra were recorded in millidegrees and normalized according to the concentration of [Zn_2_(S-MAABis)]. ^1^H NMR titration measurements were performed by adding different amounts of LeuOEt solution to a zinc bisporphyrinate solution in CDCl_3_.

### 3.2. Synthesis

#### 3.2.1. Synthesis of 5-(S-2-Methylbutanamido)-Isophthalic Acid Diamide-Linked Bisporphyrin H_4_(S-MAABis)

SOCl_2_ (10 mL) was added to 100 mL of Schlenk glass containing S-2-methylbutyric acid (0.06 g, 0.60 mmol). After 12 h of refluxing under N_2_, the excess SOCl_2_ was removed under a vacuum. The resulting white solid was dissolved in anhydrous CH_2_Cl_2_ (30 mL), after which 0.70 g of H_4_(AmBis) (0.50 mmol) and 180 μL of Et_3_N (1.3 mmol) were added. After 20 min of stirring in an ice bath, the mixture was warmed to RT. The reaction was monitored by TLC and was complete after 8 h. The solvent was removed by a rotary evaporator, and a purple solid was obtained. It was further purified by silica gel chromatography (CH_2_Cl_2_), and 0.35 g of pure product was obtained (47% yield). ^1^H NMR (400 MHz, CDCl_3_) δ 8.78 (d, 8H), 8.66 (t 4H), 8.54 (m, 4H), 8.17 (d, 4H), 8.08 (m, 12H), 7.92 (d, 2H), 7.75 (m, 6H), 7.67 (s, 12H), 7.57 (m, 4H), 7.43 (t, 2H), 7.11 (s, 2H), 6.27 (s, 1H), 5.47 (s, 2H), 3.97 (s, 1H), −0.32 (d, 2H), −0.70 (d, 3H), −0.80 (d, 1H), −0.97 (d, 3H), and −2.98 (s, 4H). ^13^C NMR (101 MHz, CDCl_3_) δ 172.69, 163.39, 142.00, 141.63, 138.26, 136.92, 135.28, 134.62, 134.35, 131.99, 129.53, 127.91, 127.81, 126.69, 123.20, 121.07, 121.00, 120.61, 118.32, 112.68, 41.86, 31.62, 25.57, 22.69, 15.79, 14.16, 10.18. IR (KBr disc, cm^−1^): 2959 (m), 2920 (m), 2851 (m), 1681 (m), 1597 (w), 1579 (w), 1552 (w), 1511 (m), 1464 (m), 1441 (m), 1348 (w), 1300 (w), 1259 (m), 1220 (w), 1178 (w), 1152 (w), 1071 (m), 1019 (m), 964 (m), 876 (w), 798 (s), 750 (m), 726 (s), 699 (s), 656 (s), 639 (s) and 557 (m). UV–vis (CH_2_Cl_2_): λ_max_ (logε) 415 (5.86), 511 (4.86), 547 (3.97), 588 (3.79), 642 (3.27). MALDI TOF-MS: *m*/*z* calcd for C_102_H_77_N_11_O_3_ 1488.59; found 1488.55 [M]^+^. Anal. Calcd for C_102_H_77_N_11_O_3_: C, 81.41; H, 5.16; N, 10.24. Found: C, 81.43; H, 5.18; N, 10.21.

#### 3.2.2. Synthesis of Zinc Bisporphyrinate [Zn_2_(S-MAABis)]

Zn(OAc)_2_·2H_2_O (0.17 g, 0.80 mmol) was added to a solution of H_4_(S-MAABis) (0.35 g, 0.23 mmol) in a mixture of CH_2_Cl_2_ and CH_3_OH (60 mL, 2:1). After 3 h of reflux, the mixture was washed with water. Then, the organic layer was collected. A purple solid was obtained after rotary evaporation. Then, it was purified by silica gel chromatography (CH_2_Cl_2_/petroleum ether = 1:1) (0.30 g, yield 82%). ^1^H NMR (400 MHz, CHCl_3_) δ 8.87 (d, 4H), 8.82 (s, 4H), 8.57 (t, 6H), 8.37 (m, 4H), 8.17 (s, 4H), 8.08 (m, 8H), 7.72 (dd, 10H), 7.61 (s, 10H), 7.45 (m, 8H), 7.01 (s, 2H), 5.12 (s, 1H), 5.01 (s, 2H), 3.66 (s, 1H), −0.31 (m, 1H), −0.80 (m, 1H), −1.14 (t, 3H), −1.33 (d, 3H), −3.58 (dd, 1H). ^13^C NMR (101 MHz, CDCl_3_) δ 172.31, 149.46, 149.23, 149.17, 149.11, 149.04, 148.14, 142.97, 141.97, 133.85, 133.54, 133.42, 133.42, 132.76, 132.29, 132.18, 131.56, 131.10, 130.96, 129.03, 128.05, 126.35, 126.21, 125.67, 125.58, 125.37, 124.49, 122.37, 120.49, 120.03, 118.81, 111.01, 30.85, 27.99, 21.66 14.41, 13.09, 8.40. IR (KBr disc, cm^−1^): 3054 (m), 2963 (m), 2928 (m), 1810 (w), 1666 (m), 1597 (m), 1581 (m), 1519 (s), 1488 (m), 1443 (m), 1340 (m), 1304 (w), 1203 (w), 1179 (w), 1156 (w), 1070 (w), 997 (s), 794 (m), 755 (m), 717 (m), 701 (m), 657 (w) and 569 (w). UV–vis (CH_2_Cl_2_): λ_max_ (logε) 419 (5.83), 553 (4.63), 592 (4.14). MALDI TOF-MS: *m*/*z* calcd for C_102_H_73_N_11_O_3_Zn_2_ 1616.43; found 1616.32 [M]^+^. Anal. Calcd for C_102_H_73_N_11_O_3_Zn_2_: C, 75.09; H, 4.51; N, 9.44. Found: C, 75.12; H, 4.48; N, 9.46.

#### 3.2.3. Synthesis and Crystal Growth of Zinc Bisporphyrinate [Zn_2_(MAABis)]

Racemic [Zn_2_(MAABis)] was synthesized via the same method as [Zn_2_(S-MAABis)]. [Zn_2_(MAABis)] (4 mg) was dissolved in CH_2_Cl_2_ (1.5 mL), and the solution was filtered and transferred to NMR test tubes. n-Hexane was added as a nonsolvent at room temperature. After 18 days, purple crystals suitable for X-ray diffraction were obtained.

### 3.3. Computation Methods

Theoretical studies on the 1:1 and 1:2 host–guest complexes formed between [Zn_2_(S-MAABis)] and l-LeuOEt were carried out. First, conformational searches were performed by HyperChem 8.0 software [29]. Then, these conformations were optimized by the PM6 method and DFT/WB97XD (3-21G basis set) method using the Gaussian 09 suite of programs [30]. Finally, DFT/WB97XD (6-31G** basis set) calculations were carried out for the 10 conformations with the lowest energies to obtain the most energetically favorable conformation for [Zn_2_(S-MAABis)(l-LeuOEt)] and [Zn_2_(S-MAABis)(l-LeuOEt)_2_]. The corresponding circular dichroism spectra were also calculated by TD-DFT for the optimized structures.

### 3.4. Single-Crystal X-ray Crystallography

Single crystals of [Zn_4_(MAABis)_2_(H_2_O)] were crystallized by diffusing n-hexane into a CH_2_Cl_2_ solution of [Zn_2_(MAABis)]. The crystallographic data of the sample were collected on a Bruker D8 VENTURE MetalJet PHOTON II diffractometer. φ/ω scanning was performed with a Ga-Kα graphite monochromator (λ = 1.34139 Å), and the crystals were maintained at 193 K during data collection. Using Olex2 [31], the structure was solved with the SHELXT [32] structure solution program using intrinsic phasing and refined with the SHELXL [33] refinement package using least squares minimization.

The crystallographic data have been deposited in the Cambridge Crystallographic Data Center (CCDC) under supplementary publication number 2367225. These data can be obtained free of charge, either from the CCDC via https://www.ccdc.cam.ac.uk/structures or from the Supporting Information. A summary of the key crystallographic data is listed in Table 3.

## 4. Conclusions

We designed and synthesized a zinc bisporphyrinate [Zn_2_(S-MAABis)], with S-2-methylbutyramido as a substituent in the linker. We investigated its ability to sense and recognize amino acid esters. Our studies suggest that the amido in the substituent is coordinated with the zinc in the host [Zn_2_(S-MAABis)] and its 1:1 host–guest complex. The corresponding 1:1 complex showed the same chirality as the 1:2 complex. This is different from [Zn_2_(AmBis)], which exhibited chiral inversion during titration. The theoretical calculations indicated that the amido groups in the 1:1 host–guest complex [Zn_2_(S-MAABis)(l-LeuOEt)] participated not only in coordination, but also in hydrogen bonds. However, the amino groups in [Zn_2_(AmBis)(l-LeuOEt)] only participated in coordination, which led to completely different conformations of the bisporphyrins in the two structures, corresponding to different chiralities. Therefore, this work suggested that the introduction of coordination sites affects the chirality transfer process, but does not always lead to chirality inversion. The supramolecular chirality is dependent on the specific binding mode.

On the other hand, the introduction of chiral groups results in chiral [Zn_2_(S-MAABis)]. However, our results indicate that its enantioselectivity toward amino acid esters is quite weak, which may be due to the chiral substituent being far from the ligated amino acid esters. Our future plan is to design chiral bisporphyrin molecules with chiral coordination sites closer to the porphyrin center to achieve controllable chiral induction and transfer.

## Figures and Tables

**Table 1:** CD spectral data of the complexes of [Zn_2_(S-MAABis)] with amino acid esters in a mixed solvent (n-hexane:dichloromethane = 4:1) at 298 K.

Amino Acid Esters	[Zn_2_(S-MAABis)]		Amino Acid Esters	[Zn_2_(S-MAABis)]	
	Δε (cm^−1^M^−1^), λ (nm)	A_obs_^a^		Δε (cm^−1^M^−1^), λ (nm)	A_obs_^a^
l-LeuOEt	796, 421	−1861	d-LeuOEt	−748, 421	1754
	−1065, 430			1006, 430	
l-ValOEt	996, 421	−2269	d-ValOEt	−775, 421	1827
	−1273, 431			1052, 431	
l-AlaOEt	677, 421	−1475	d-AlaOEt	−643, 421	1480
	−798, 430			837, 430	
l-PhgOEt	740, 421	−1726	d-PhgOEt	−704, 421	1646
	−986, 431			942, 431	
l-PheOEt	932, 421	−2164	d-PheOEt	−919, 421	2149
	−1232, 431			1230, 431	

^a^ A_obs_ = Δε1 − Δε2, cm^−1^M^−1.^

**Table 2:** Formation constants (K_f_) and enantioselectivities (α) of [Zn_2_(S-MAABis)] toward amino acid esters according to UV–vis titration data.

	K_1_	K_2_	K_f_	α
d-LeuOEt	8.7 × 10^4^	5.8 × 10^2^	5.0 × 10^7^	0.81
l-LeuOEt	5.2 × 10^4^	1.2 × 10^3^	6.2 × 10^7^	
d-ValOEt	5.2 × 10^4^	4.0 × 10^2^	2.1 × 10^7^	1.9
l-ValOEt	2.4 × 10^4^	4.7 × 10^2^	1.1 × 10^7^	
d-AlaOEt	2.6 × 10^4^	1.0 × 10^3^	2.6 × 10^7^	0.93
l-AlaOEt	2.9 × 10^4^	9.8 × 10^2^	2.8 × 10^7^	
d-PhgOEt	6.4 × 10^4^	3.1 × 10^2^	2.0 × 10^7^	0.74
l-PhgOEt	3.7 × 10^4^	7.2 × 10^2^	2.7 × 10^7^	
d-PheOEt	1.8 × 10^5^	3.1 × 10^2^	5.6 × 10^7^	1.8
l-PheOEt	9.4 × 10^4^	3.3 × 10^2^	3.1 × 10^7^	

K_1_ and K_2_ are the equilibrium constants for equilibria 1 and 2, respectively; K_f_ is the overall formation constant for the 1:2 host–guest complexes; and α = K_f(D)_/K_f(L)_.

**Table 3:** Crystal data and structure refinement parameters for [Zn_4_(MAABis)_2_(H_2_O)].

	[Zn_4_(MAABis)_2_(H_2_O)]
CCDC number	2367225
Formula	C_202_H_140_N_22_O_7_Zn_4_
Formula weight	3248.83
Crystal system	monoclinic
Space group	*P*2_1_/*n*
*a*/Å	24.5519(19)
*b*/Å	24.7223(17)
*c*/Å	33.671(2)
*α*/°	90
*β*/°	104.566(4)
*γ*/°	90
*V*/Å^3^	19,781(2)
*Z*	4
*D_c_*/(g cm^−3^)	1.091
*F*(000)	6728.0
μ/mm^−1^	0.650
Crystal size/mm^3^	0.12 × 0.1 × 0.09
Radiation/Å	GaKα (λ = 1.34139)
2θ range for data collection/°	3.492 to 104.194
Index ranges	−28 ≤ h ≤ 28, −28 ≤ k ≤ 29, −23 ≤ l ≤ 39
Reflections collected	141611
Independent reflections	33621 [R_int_ = 0.0824, R_sigma_ = 0.0884]
Data/restraints/parameters	33621/1631/1906
Goodness-of-fit on F^2^	1.070
Final R indexes [I ≥ 2σ (I)]	R_1_ = 0.1170, wR_2_ = 0.2915
Final R indexes [all data]	R_1_ = 0.2050, wR_2_ = 0.3375
Largest diff. peak/hole/e Å^−3^	0.59/−0.68

R_1_ = ∑(||F_0_| − |F_c_||)/∑|F_0_|, wR_2_ = ∑w(|F_0_|^2^ − |F_c_|^2^)^2^/∑w(|F_0_|^2^)^2^]^1/2^.

## Data Availability

The original contributions presented in the study are included in the article/Supplementary Material, further inquiries can be directed to the corresponding authors.

## Supplementary Materials

The following supporting information can be downloaded at https://www.mdpi.com/article/10.3390/molecules29153652/s1. Figure S1: _1_H-^1^H COSY spectrum of H_4_(S-MAABis); Figure S2: ^1^H-^1^H COSY spectrum of [Zn_2_(S-MAABis)]; Figure S3: ^1^H NMR spectrum of 2-methylbutyric acid; Figure S4: Intermolecular hydrogen bonds [Zn_4_(MAABis)_2_(H_2_O)]; Figures S5–S9: CD spectra of [Zn_2_(S-MAABis)] in the presence of a large excess of amino acid esters; Figures S10–S13: CD titration spectra; Figure S14: ^1^H NMR titration spectra; Figures S15–S23: UV–vis titration spectra; Cartesian coordinates for optimized structure of [Zn_2_((S-MAABis)(l-LeuOEt)]; Cartesian coordinates for optimized structure of [Zn_2_((S-MAABis)(l-LeuOEt)_2_].
