# Supplementary material for: Chirality Sensing of Amino Acid Esters by S-2-Methylbutanamido-Substituted m-Phthalic Diamide-Linked Zinc Bisporphyrinate
Source: Molecules. 2024 Aug 1;29(15):3652. doi: 10.3390/molecules29153652 (PMC11314088; doi:10.3390/molecules29153652)
Supplement: Supplementary file 1 [file molecules-29-03652-s001.zip › molecules-3122812-supplementary.pdf]

Supporting Information

Chirality Sensing of Amino Acid Esters by S-2-Methylbutanamido-Substituted *m*-Phthalic Diamide-linked Zinc Bisporphyrinate

Zhipeng Li, Yue Zhao, Yong Wang, Wen-Hua Zhang* and Chuanjiang Hu*

Figure S1. ^{1}H-^{1}H COSY spectrum of H_4(S-MAABis)..S2

Figure S2. ^{1}H-^{1}H COSY spectrum of [Zn_2(S-MAABis)]....................................S3

Figure S3. ^{1}H NMR spectrum of S-2-methylbutyric acid..................................S4

Figure S4. Intermolecular hydrogen bonds in [Zn_4(MAABis)$_2$(H_2O)].........S5

Figures S5-S9. CD spectra of [Zn_2(S-MAABis)] before and after the addition of a large excess of amino acid esters S6

Figures S10-S13. CD titration spectra S11

Figure S14. ^{1}H NMR titration spectra S14

Figures S15-S23. UV-Vis titration spectra...S15

Cartesian coordinates for optimized structure of [Zn_2((S-MAABis)(L-LeuOEt)]....S25

Cartesian coordinates for optimized structure of [Zn_2((S-MAABis)(L-LeuOEt)$_2$]...S31

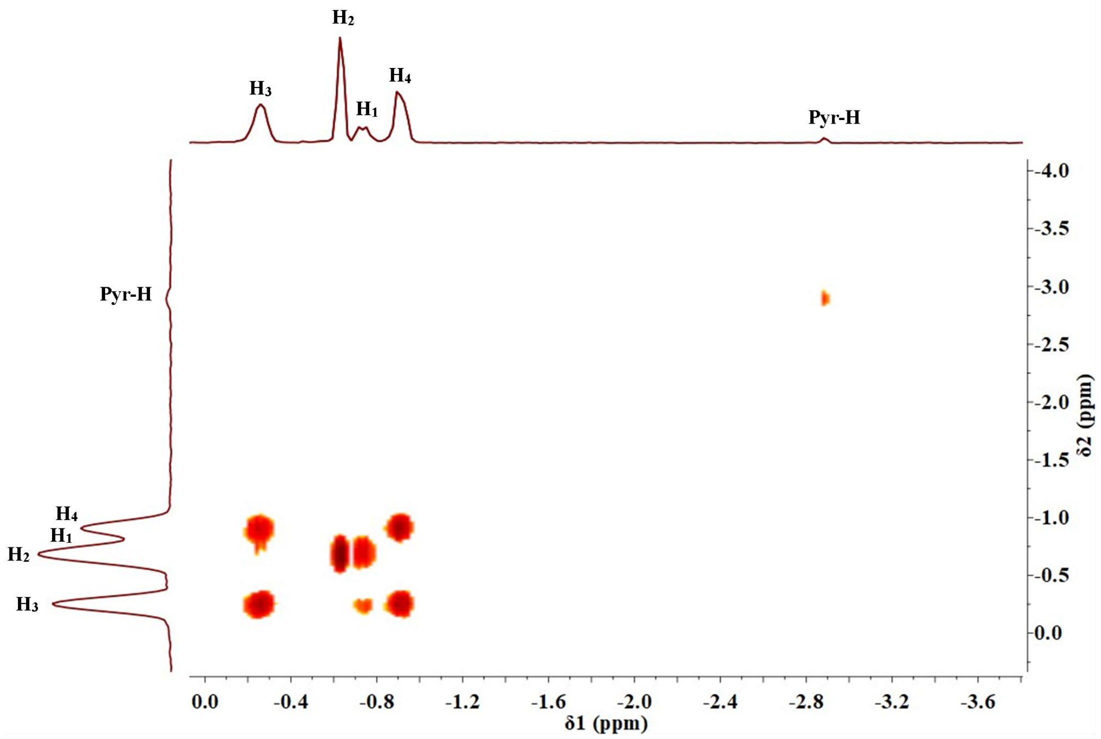

Figure S1. ${}^{1}H$-${}^{1}H$ COSY spectrum of H_4(S-MAABis) in $CDCl_3$ (6.1×10^{-4} M).

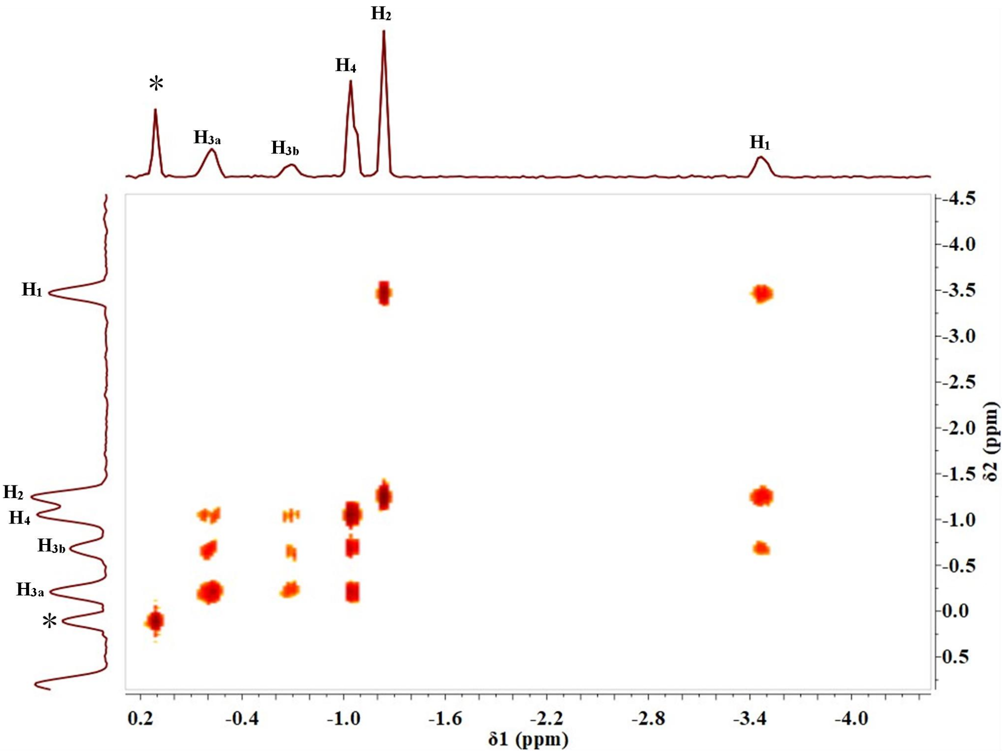

Figure S2. ^{1}H-^{1}H COSY spectrum of [Zn_2(S-MAABis)] in $CDCl_3$ (6.1×10^{-4} M). *TMS.

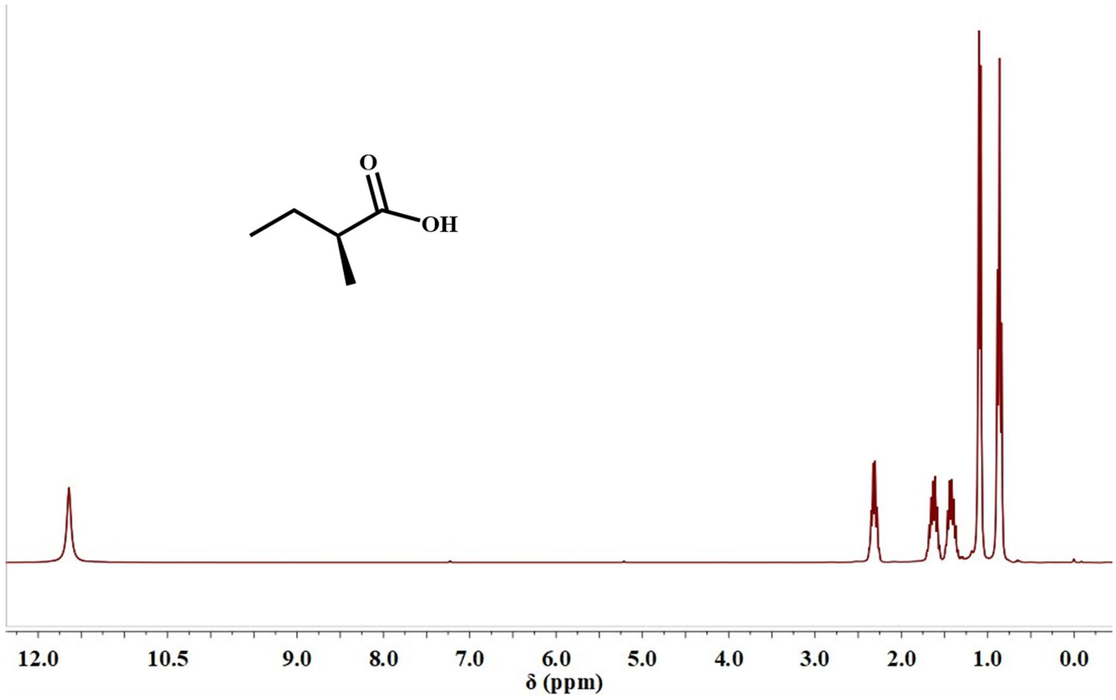

Figure S3. ^{1}H NMR spectrum of S-2-methylbutyric acid in $CDCl_3$ (8.0×10^{-2} M).

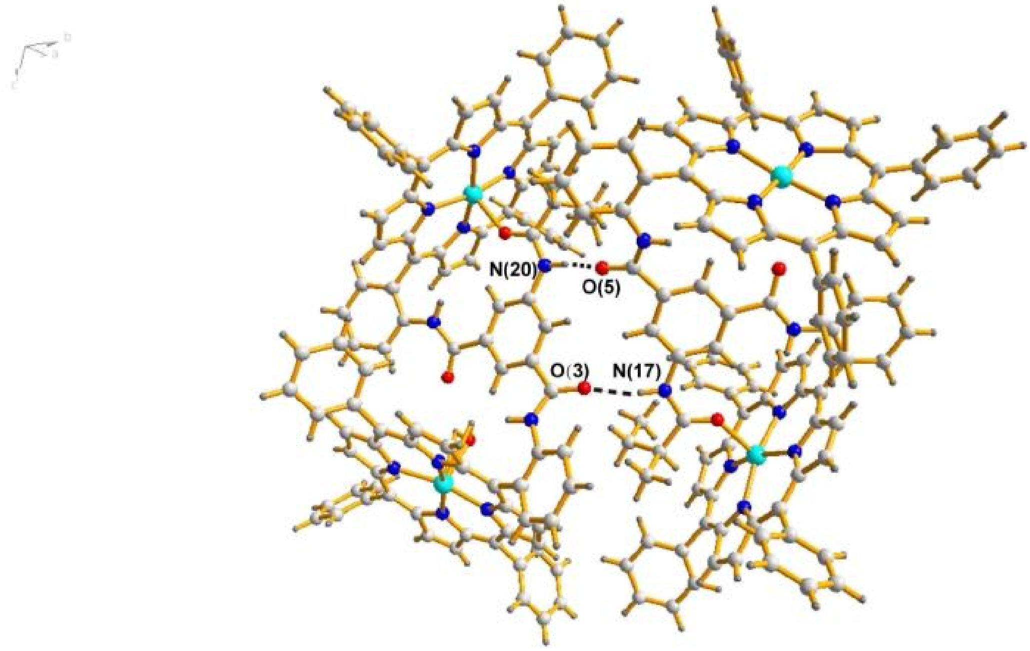

Figure S4. Intermolecular hydrogen bonds between Mol **1** and Mol **2 in** [$Zn_4(MAABis)_2(H_2O)$]. N(20)···O(5) = 3.024 Å. O(3) ···· N(17) = 2.974 Å.

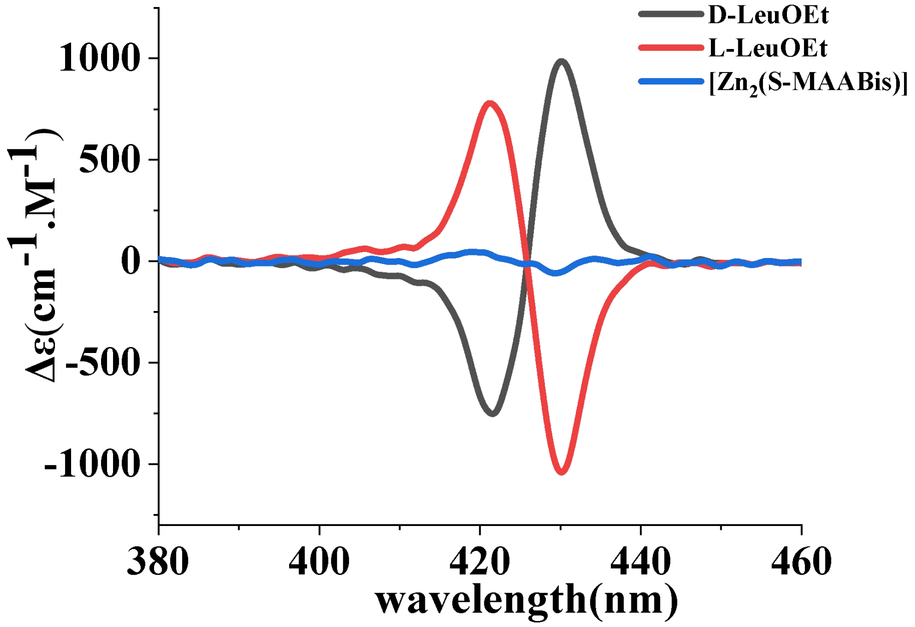

Figure S5. CD spectra of [Zn_2(S-MAABis)] (1.0×10^{-6} M) before (blue) and after the addition of a large excess (1000 equivalents) of LeuOEt (red, L-enantiomer; black, D-enantiomer).

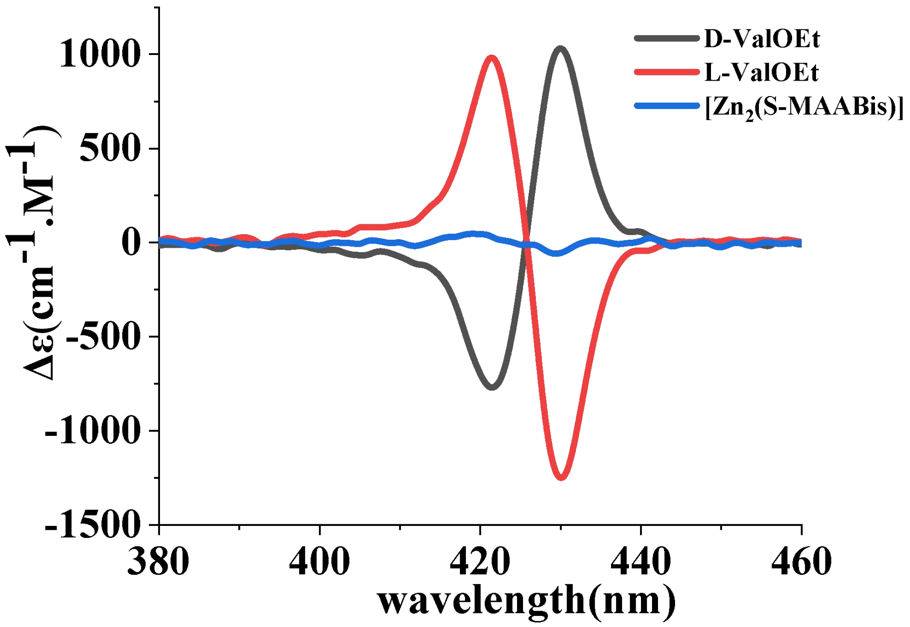

Figure S6. CD spectra of $[Zn_2(S\text{-}MAABis)]$ (1.0×10^{-6} M) before (blue) and after the addition of a large excess (1000 equivalents) of ValOEt (red, L-enantiomer; black, D-enantiomer).

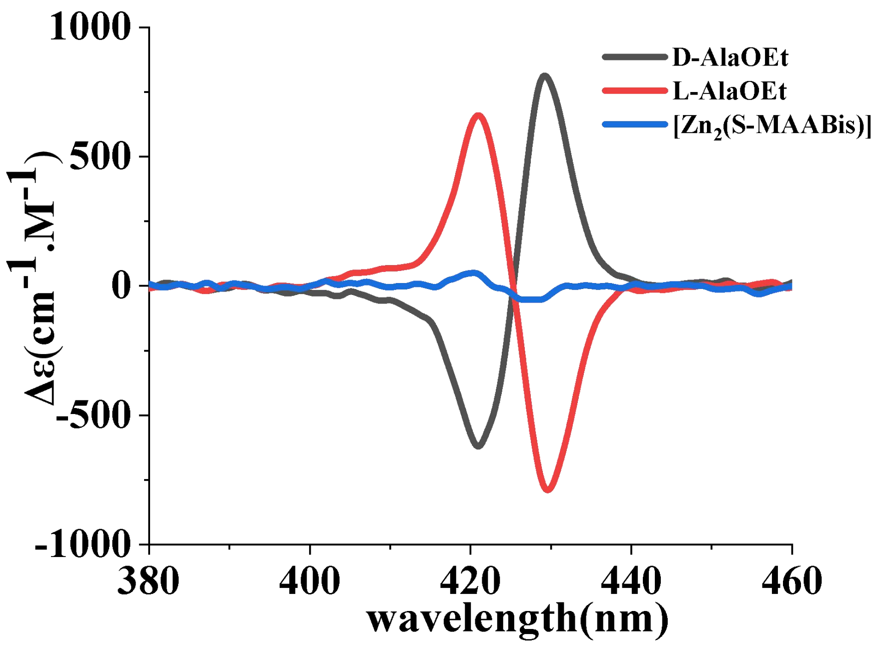

Figure S7. CD spectra of [Zn_2(S-MAABis)] (1.0×10^{-6} M) before (blue) and after the addition of a large excess (1000 equivalents) of AlaOEt (red, L-enantiomer; black, D-enantiomer).

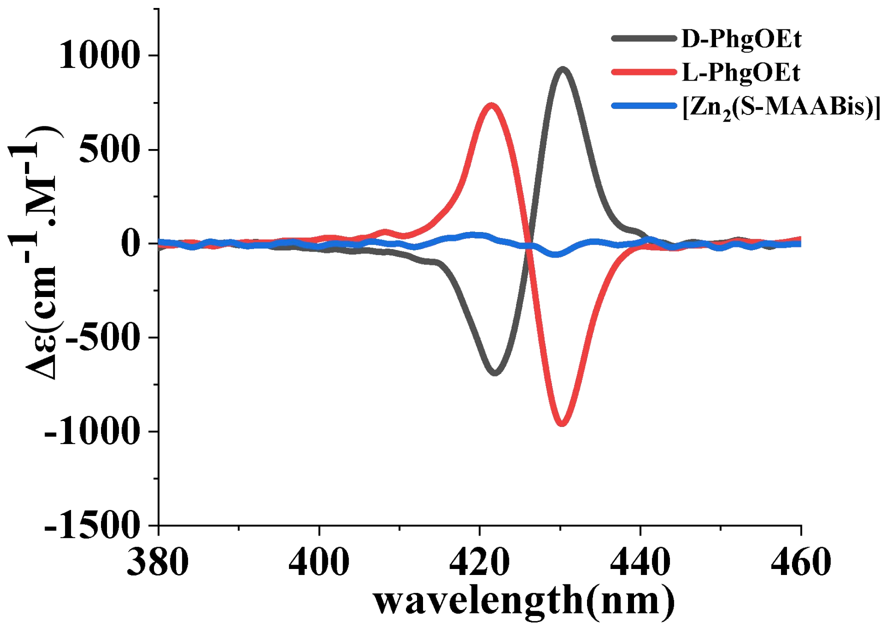

Figure S8. CD spectra of [Zn_2(S-MAABis)] (1.0×10^{-6} M) before (blue) and after the addition of a large excess (1000 equivalents) of PhgOEt (red, L-enantiomer; black, D-enantiomer).

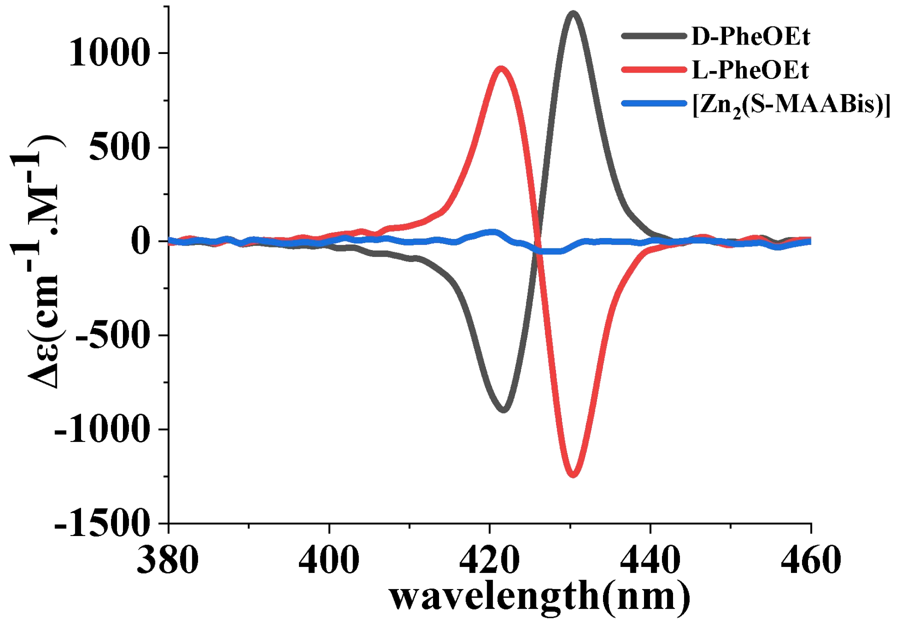

Figure S9. CD spectra of [Zn_2(S-MAABis)] (1.0×10^{-6} M) before (blue) and after the addition of a large excess (1000 equivalents) of PheOEt (red, L-enantiomer; black, D-enantiomer).

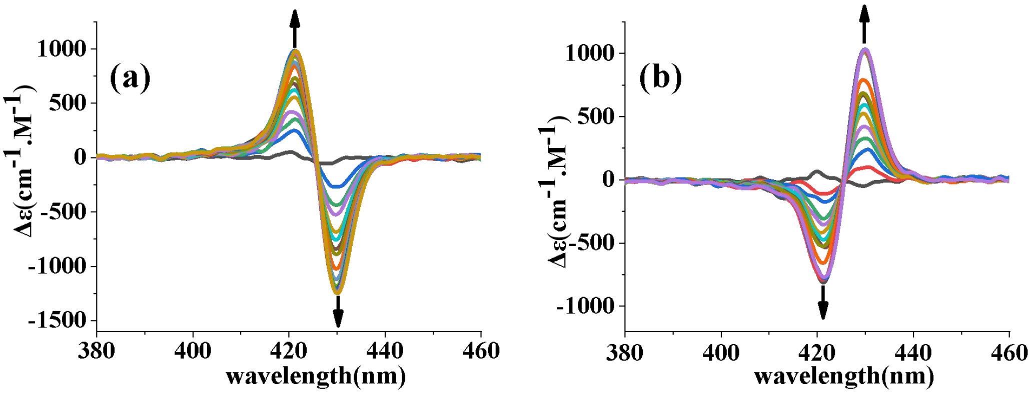

Figure S10. CD spectra of a mixture of [Zn_2(S-MAABis)] (1.0 × 10^{-6} M) and 0-1000 equivalents of guest (a) L-ValOEt and (b) D-ValOEt in a mixed solvent (n-hexane: dichloromethane=4:1) at 298 K.

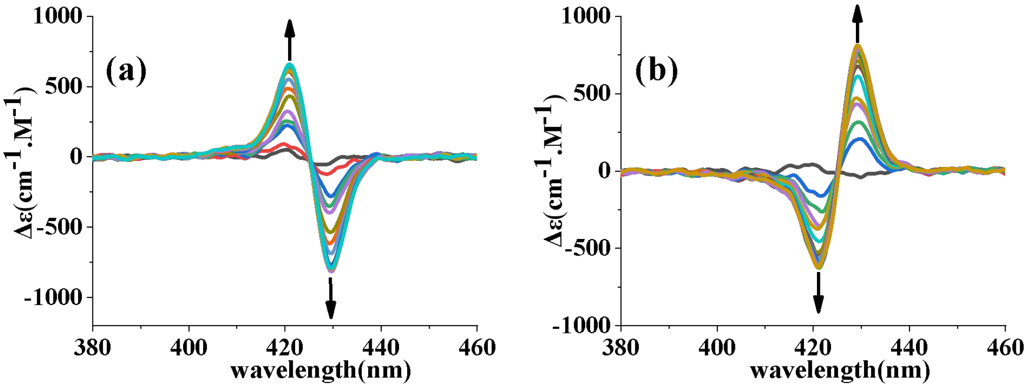

Figure S11. CD spectra of a mixture of [Zn_2(S-MAABis)] (1.0×10^{-6} M) and 0-1000 equivalents of guest (a) L-AlaOEt and (b) D-AlaOEt in a mixed solvent (n-hexane: dichloromethane=4:1) at 298 K.

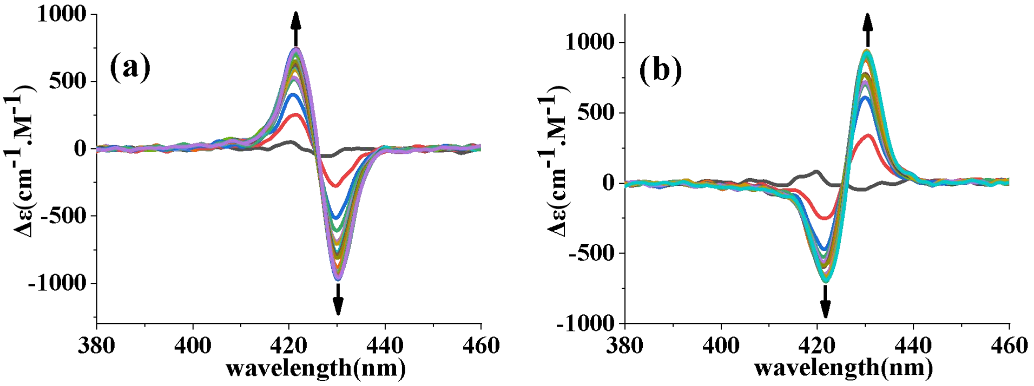

Figure S12. CD spectra of a mixture of [Zn_2(S-MAABis)] (1.0×10^{-6} M) and 0-1000 equivalents of guest (a) L-PhgOEt and (b) D-PhgOEt in a mixed solvent (n-hexane: dichloromethane=4:1) at 298 K.

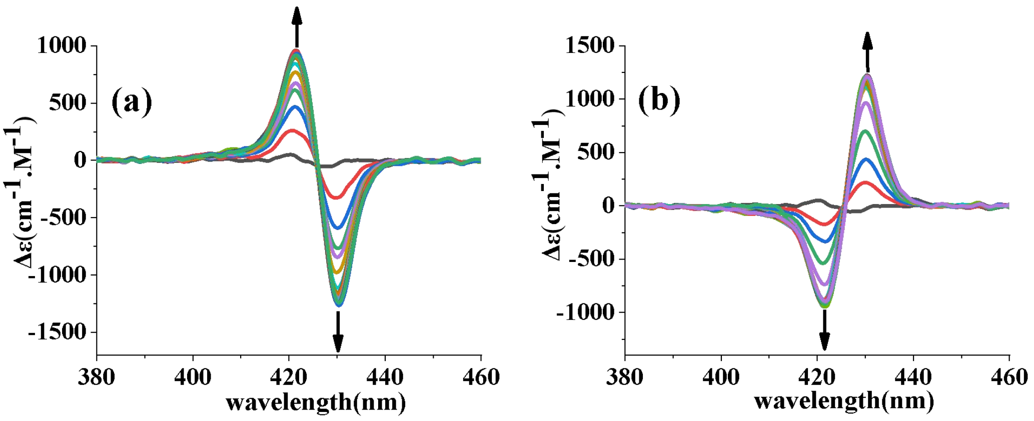

Figure S13. CD spectra of a mixture of [Zn_2(S-MAABis)] (1.0×10^{-6} M) and 0-1000 equivalents of guest (a) L-PheOEt and (b) D-PheOEt in a mixed solvent (n-hexane: dichloromethane=4:1) at 298 K.

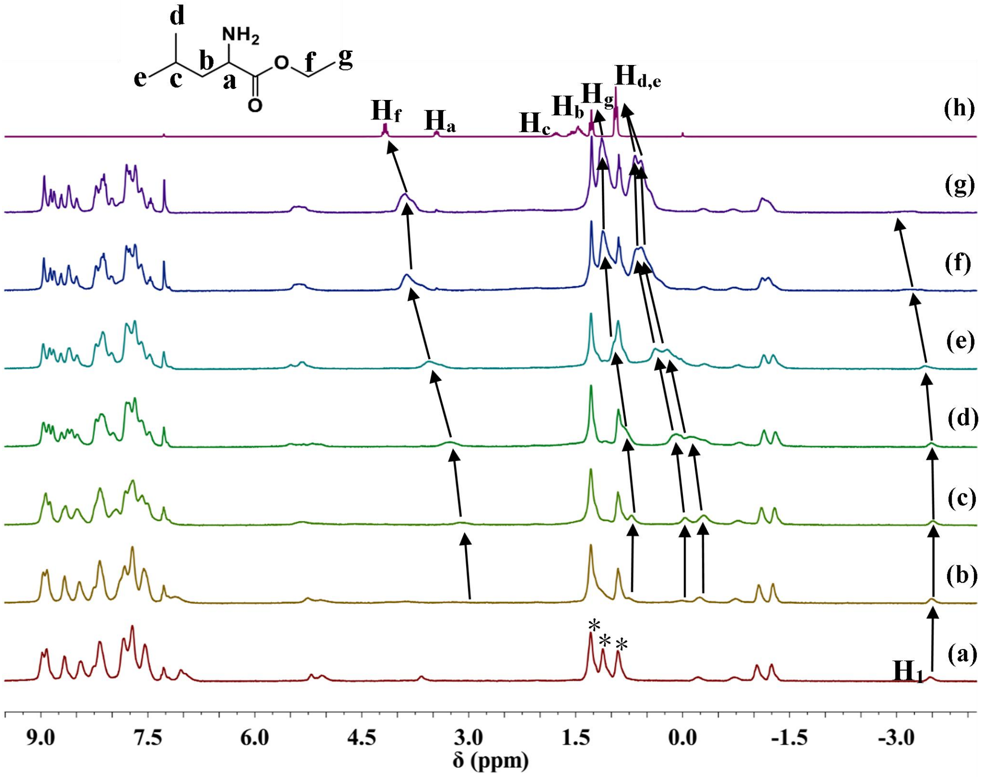

Figure S14. ^{1}H NMR spectra of [Zn_2(S-MAABis)] in $CDCl_3$ (6.0×10^{-3} M) with D-LeuOEt at 298 K. The equivalents of L-leucine ethyl ester are (A) 0 eq., (B) 0.4 eq., (C) 0.8 eq., (D) 1.0 eq., (E) 1.8 eq., (F) 2.2 eq., (G) 3.2 eq., and (H) L-LeuOEt. *Impurities, such as water and petroleum ether.

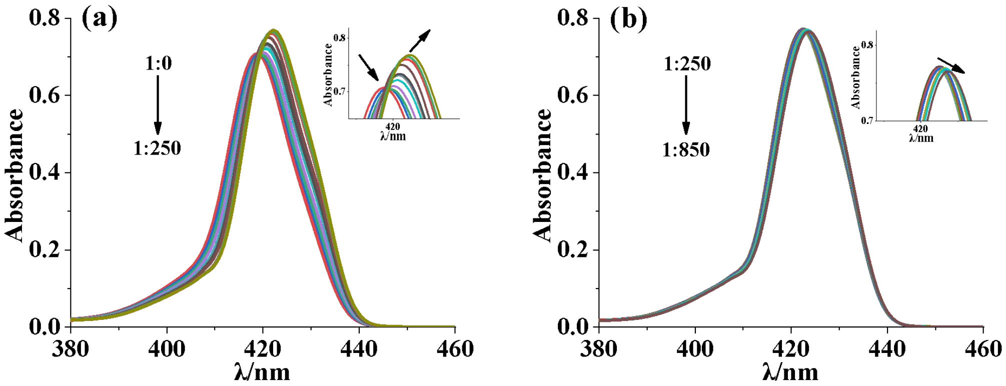

Figure S15. UV–visible titration spectra of a mixture of [Zn_2(S-MAABis)] (1.0×10^{-6} M) and D-LeuOEt in a mixed solvent (n-hexane:dichloromethane=4:1) at 298 K, with host-guest ratios ranging from (a) 1:0 to 1:250 and (b) 1:250 to 1:850.

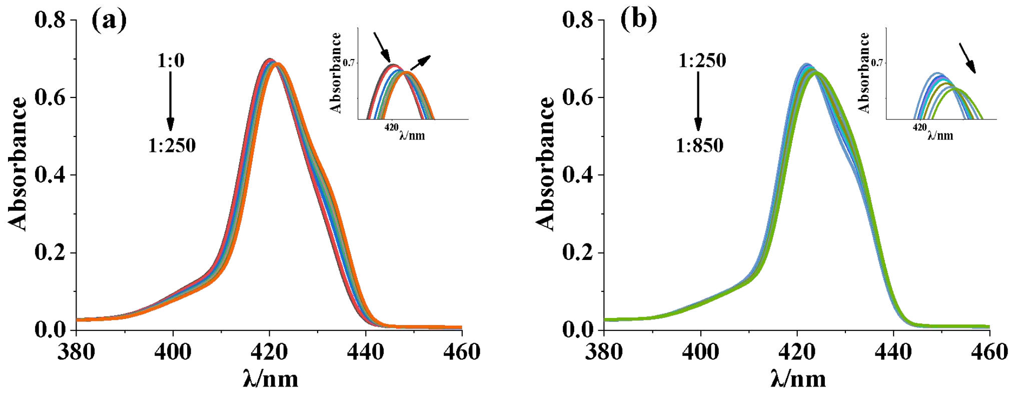

Figure S16. UV–visible titration spectra of a mixture of [Zn_2(S-MAABis)] (1.0×10^{-6} M) and L-ValOEt in a mixed solvent (n-hexane:dichloromethane=4:1) at 298 K, with host-guest ratios ranging from (a) 1:0 to 1:250 and (b) 1:250 to 1:850.

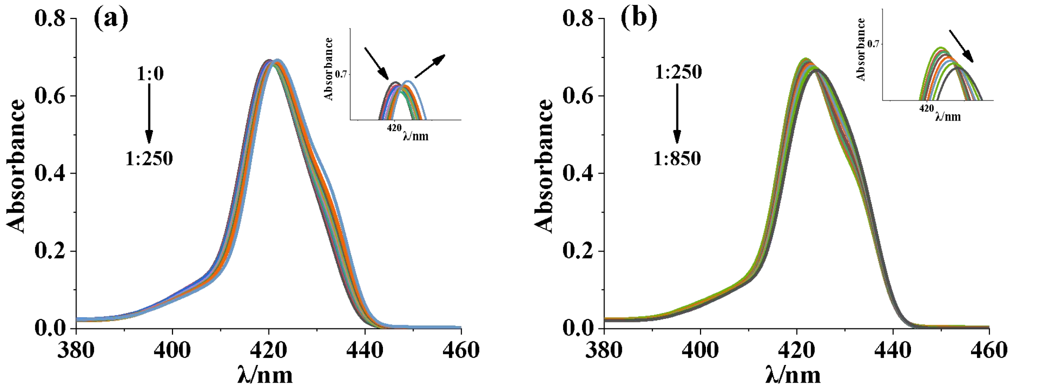

Figure S17. UV–visible titration spectra of a mixture of [Zn_2(S-MAABis)] (1.0×10^{-6} M) and D-ValOEt in a mixed solvent (n-hexane:dichloromethane=4:1) at 298 K, with host-guest ratios ranging from (a) 1:0 to 1:250 and (b) 1:250 to 1:850.

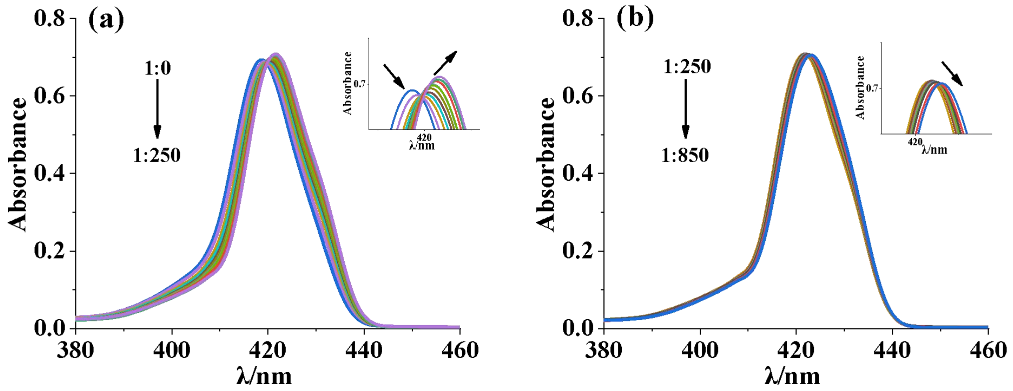

Figure S18. UV–visible titration spectra of a mixture of [Zn_2(S-MAABis)] (1.0×10^{-6} M) and L-AlaOEt in a mixed solvent (n-hexane:dichloromethane=4:1) at 298 K, with host-guest ratios ranging from (a) 1:0 to 1:250 and (b) 1:250 to 1:850.

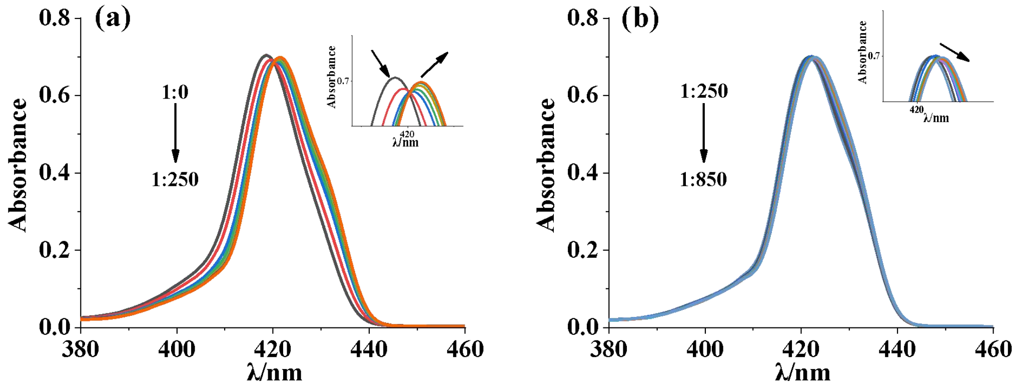

Figure S19. UV–visible titration spectra of a mixture of [Zn_2(S-MAABis)] (1.0 × 10^{-6} M) and D-AlaOEt in a mixed solvent (n-hexane:dichloromethane=4:1) at 298 K, with host-guest ratios ranging from (a) 1:0 to 1:250 and (b) 1:250 to 1:850.

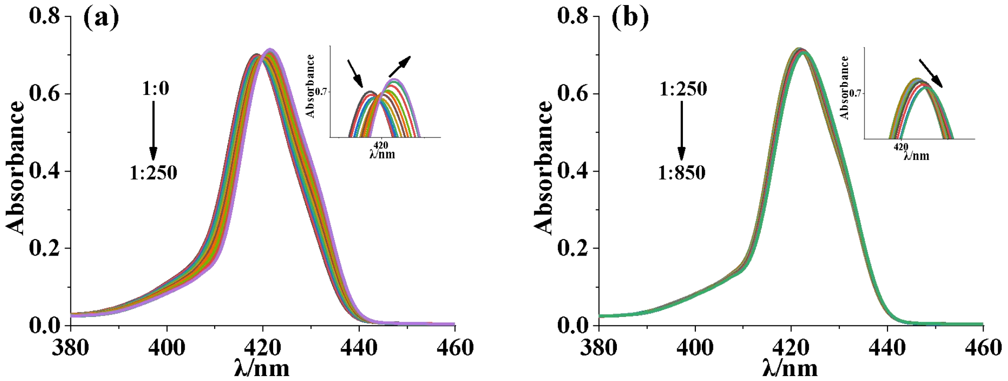

Figure S20. UV–visible titration spectra of a mixture of [Zn_2(S-MAABis)] (1.0 × 10^{-6} M) and L-PhgOEt in a mixed solvent (n-hexane:dichloromethane=4:1) at 298 K, with host-guest ratios ranging from (a) 1:0 to 1:250 and (b) 1:250 to 1:850.

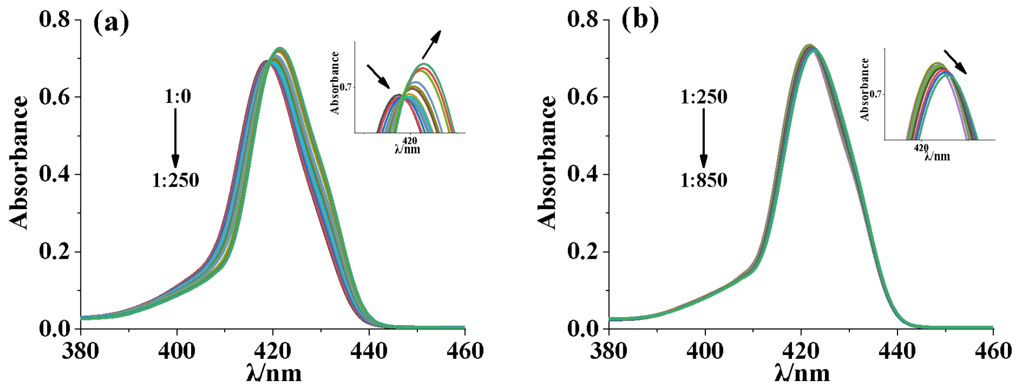

Figure S21. UV–visible titration spectra of a mixture of [Zn_2(S-MAABis)] (1.0×10^{-6} M) and D-PhgOEt in a mixed solvent (n-hexane:dichloromethane=4:1) at 298 K, with host-guest ratios ranging from (a) 1:0 to 1:250 and (b) 1:250 to 1:850.

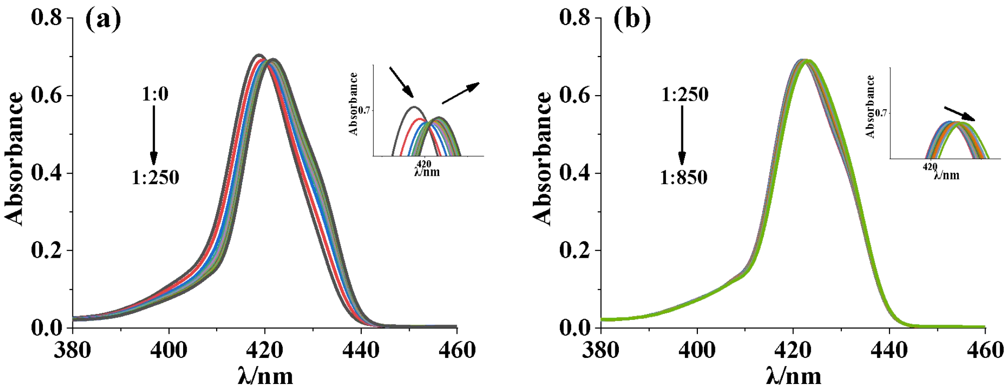

Figure S22. UV–visible titration spectra of a mixture of [Zn_2(S-MAABis)] (1.0×10^{-6} M) and L-PheOEt in a mixed solvent (n-hexane:dichloromethane=4:1) at 298 K, with host-guest ratios ranging from (a) 1:0 to 1:250 and (b) 1:250 to 1:850.

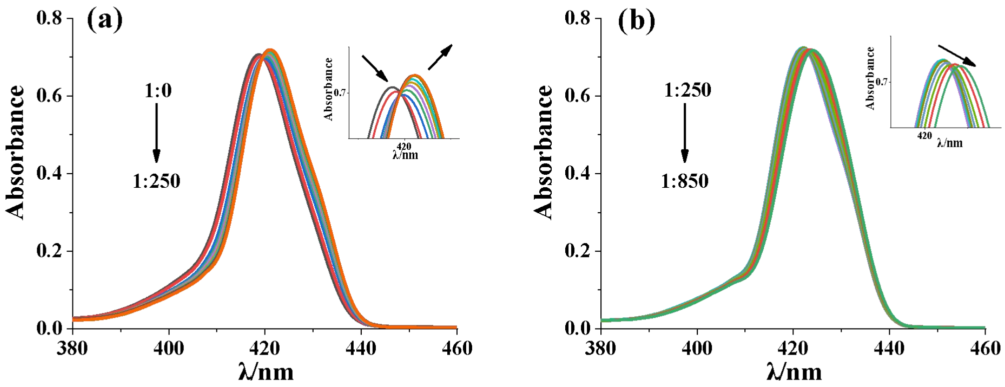

Figure S23. UV–visible titration spectra of a mixture of [Zn_2(S-MAABis)] (1.0×10^{-6} M) and D-PheOEt in a mixed solvent (n-hexane:dichloromethane=4:1) at 298 K, with host-guest ratios ranging from (a) 1:0 to 1:250 and (b) 1:250 to 1:850.

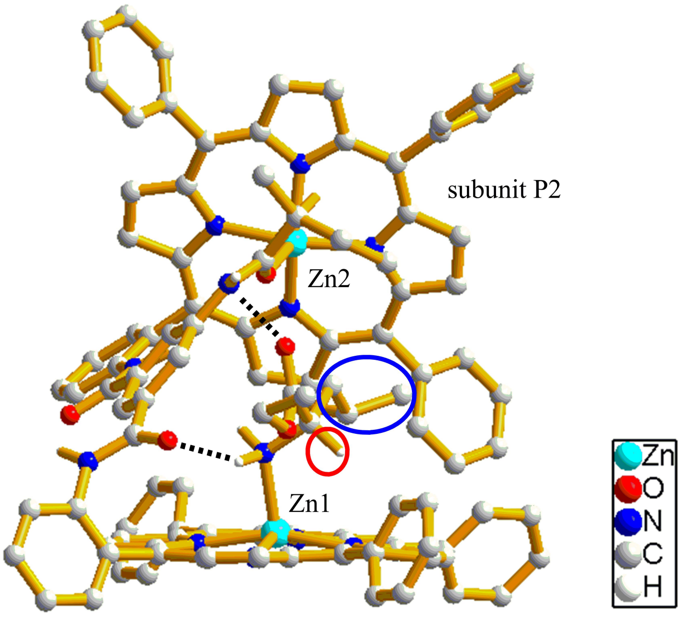

[Zn_2(S-MAABis)(L-LeuOEt)]

ΔE^{θ} =-8525.1525517 Hartree

Cartesian coordinates for Geometry Optimized [Zn_2(S-MAABis)(L-LeuOEt)]

Atoms	x	y	z
Zn	-3.996300	0.253100	0.615700
Zn	4.326400	0.133400	0.271200
O	-3.535200	-2.279200	-3.124700
O	2.641100	-0.346200	-1.009100
O	-0.302700	-5.295100	1.311600
N	-5.535700	-0.066800	-0.787300
N	-4.430100	2.268200	0.517000
N	-3.122000	0.584200	2.446400
C	-2.567100	-0.370600	3.250300
C	-1.899100	0.256900	4.364200
H	-1.387600	-0.258200	5.162600
C	-2.063000	1.594100	4.216800
H	-1.704300	2.370400	4.874300
C	-2.840200	1.793900	3.018000
N	-4.114900	-1.761200	1.082300
C	-4.720900	-2.733300	0.342800
C	-4.401800	-4.032500	0.888700
H	-4.771100	-4.976900	0.513600

C	-3.577700	-3.826300	1.945400
H	-3.109400	-4.568800	2.573800
C	-3.415300	-2.394600	2.072100
N	5.740600	0.334300	-1.226900
N	4.399000	2.166400	0.551500
N	3.519500	-0.025000	2.168200
N	4.799400	-1.869000	0.354900
N	1.337000	-1.076300	-2.707900
H	1.009200	-0.817300	-3.628500
N	1.359000	-3.779700	1.744300
H	1.719400	-2.883900	1.449900
N	-3.687000	-4.357900	-2.197600
H	-3.149500	-5.091800	-1.762200
C	-2.128200	6.545900	4.775100
H	-1.850300	7.428700	5.342200
C	-2.955400	5.580000	5.342400
H	-3.332400	5.709700	6.352000
C	-3.312300	4.449200	4.614200
H	-3.967400	3.702800	5.052500
C	-2.843200	4.260300	3.309400
C	-2.012400	5.238000	2.752200
H	-1.638900	5.100500	1.743400
C	-1.658800	6.371000	3.477000
H	-1.003100	7.111100	3.029900
C	-3.236300	3.049800	2.526600
C	-3.986800	3.251200	1.359500
C	-4.432100	4.539900	0.881500
H	-4.257300	5.485300	1.372400
C	-5.121500	4.318300	-0.263800
H	-5.618800	5.044400	-0.890100
C	-5.127700	2.891100	-0.476900
C	-5.809700	2.259600	-1.529100
C	-5.482200	3.795400	-3.476400
H	-4.412500	3.662400	-3.346700
C	-5.971800	4.601500	-4.499100
H	-5.279700	5.106900	-5.165600
C	-7.345300	4.756100	-4.668800
H	-7.728900	5.384700	-5.466100
C	-8.223800	4.102200	-3.809600
H	-9.295600	4.222200	-3.932800
C	-7.732300	3.296500	-2.786700
H	-8.415200	2.787100	-2.113800

C	-6.356300	3.132100	-2.609700
C	-5.996900	0.879900	-1.654300
C	-6.672200	0.246500	-2.766900
H	-7.122400	0.763300	-3.601800
C	-6.599600	-1.087500	-2.557500
H	-6.970300	-1.877300	-3.193200
C	-5.877600	-1.276100	-1.315900
C	-5.516700	-2.525700	-0.795500
C	-2.672500	-1.762500	3.078800
C	-2.785000	-3.415900	4.960800
H	-3.864200	-3.422100	4.842700
C	-2.012900	-2.617600	4.110400
C	-0.625500	-2.598400	4.283300
H	-0.014000	-1.978100	3.633900
C	-0.028800	-3.354800	5.286200
H	1.049900	-3.346100	5.404800
C	-0.805200	-4.152000	6.120900
H	-0.332300	-4.748400	6.894700
C	-2.186100	-4.182200	5.954800
H	-2.801500	-4.795200	6.605900
C	-5.566300	-5.664200	-2.962600
H	-4.855900	-6.283400	-3.501400
C	-6.923200	-5.961200	-2.982800
H	-7.279700	-6.825500	-3.533000
C	-7.815400	-5.140500	-2.301300
H	-8.877800	-5.360700	-2.309500
C	-7.343000	-4.036700	-1.601000
H	-8.033500	-3.395700	-1.062300
C	-5.981200	-3.725200	-1.555200
C	-5.094400	-4.559000	-2.258500
C	-3.035800	-3.198900	-2.488400
C	0.885600	-2.628600	-0.844900
H	1.884200	-2.457000	-0.462200
C	0.494000	-1.969700	-2.015600
C	-0.760800	-2.219400	-2.565600
H	-1.080800	-1.700700	-3.462600
C	-1.644000	-3.093700	-1.937900
C	-1.269100	-3.727200	-0.759800
H	-1.944400	-4.367800	-0.204800
C	-0.006800	-3.500400	-0.219800
C	0.324600	-4.286800	1.023700
C	2.343700	-0.309700	-2.207100

C	3.051100	0.574700	-3.220100
H	3.935000	0.940900	-2.698700
C	3.515300	-0.222600	-4.444800
H	2.681500	-0.517500	-5.091900
H	4.067100	-1.120600	-4.153400
H	4.189800	0.399100	-5.038900
C	2.181800	1.793000	-3.599900
H	1.153800	1.478000	-3.818300
H	2.579500	2.208400	-4.533500
C	2.189000	2.867400	-2.515800
H	1.998700	2.435100	-1.530100
H	1.428800	3.630700	-2.702600
H	3.159500	3.365500	-2.462600
C	2.333800	-5.915400	4.622300
H	2.000000	-6.805600	5.145700
C	3.452500	-5.221800	5.070900
H	4.001300	-5.566600	5.940900
C	3.877200	-4.087400	4.383100
H	4.763700	-3.547800	4.701200
C	3.187800	-3.634300	3.262700
C	2.033600	-4.325300	2.846600
C	1.614500	-5.474800	3.516200
H	0.724600	-5.987600	3.180000
C	3.660900	-2.468500	2.450900
C	4.360400	-2.772400	1.274200
C	4.692600	-4.115100	0.850700
H	4.474100	-5.015800	1.405900
C	5.325400	-4.000800	-0.340400
H	5.736300	-4.790700	-0.951800
C	5.392500	-2.587500	-0.644100
C	6.026800	-2.051700	-1.768500
C	7.910500	-3.195600	-2.990000
H	8.612500	-2.631700	-2.383400
C	8.372700	-4.090700	-3.949500
H	9.440500	-4.224400	-4.090900
C	7.470100	-4.818100	-4.720500
H	7.831400	-5.517100	-5.468100
C	6.102600	-4.647800	-4.525600
H	5.392500	-5.212900	-5.121200
C	5.641500	-3.751500	-3.566600
H	4.574900	-3.620500	-3.407200
C	6.539400	-3.012300	-2.790500

C	6.217900	-0.680000	-2.005400
C	6.940400	-0.138600	-3.131100
H	7.421900	-0.717600	-3.905000
C	6.900300	1.211700	-3.009900
H	7.350900	1.947300	-3.659400
C	6.119800	1.500500	-1.830900
C	5.730700	2.786400	-1.431400
C	7.085000	4.878100	-1.759700
H	7.456100	4.758600	-0.746500
C	6.187200	3.935900	-2.269100
C	5.716500	4.094900	-3.576500
H	5.020400	3.364800	-3.980100
C	6.130300	5.170900	-4.354800
H	5.751800	5.281800	-5.366100
C	7.023400	6.104200	-3.836400
H	7.346800	6.944300	-4.442500
C	7.500100	5.954400	-2.537100
H	8.200100	6.675800	-2.127700
C	4.907600	3.076400	-0.334500
C	4.455900	4.402300	0.010900
H	4.682400	5.303500	-0.539100
C	3.699400	4.279900	1.128400
H	3.184200	5.061300	1.666000
C	3.689200	2.878600	1.477000
C	3.062800	2.351700	2.621800
C	2.778400	5.205400	5.099000
H	3.430900	5.948900	5.546100
C	3.299700	4.288100	4.192900
H	4.354400	4.315800	3.937300
C	2.481500	3.318400	3.601000
C	1.130500	3.277800	3.962500
H	0.480900	2.523700	3.530200
C	0.608000	4.196900	4.865500
H	-0.448600	4.174700	5.112100
C	1.427900	5.167100	5.432200
H	1.012200	5.888200	6.128800
C	3.015400	0.988900	2.935700
C	2.451000	0.449500	4.153800
H	2.009700	1.028800	4.950300
C	2.611100	-0.892800	4.099000
H	2.329800	-1.623900	4.841700
C	3.290500	-1.180900	2.857100

N	-2.612300	-0.033700	-1.012600
H	-1.833600	-0.650700	-0.797200
H	-3.234000	-0.574600	-1.617200
C	-2.116300	1.147400	-1.751300
H	-2.833600	1.956000	-1.572000
C	-2.156200	0.839400	-3.243800
C	-0.738200	1.575700	-1.277800
H	-0.468700	2.470000	-1.847300
H	-0.009100	0.809400	-1.559400
C	-0.672500	1.861500	0.237900
H	-1.698200	1.991500	0.593800
C	0.068700	3.167100	0.526000
H	-0.004100	3.430100	1.583500
H	1.133200	3.093000	0.283300
H	-0.353600	3.994100	-0.056400
C	-0.049500	0.698300	1.017400
H	-0.598900	-0.238100	0.869900
H	0.989000	0.533000	0.710400
H	-0.060700	0.897300	2.093300
C	-3.711500	0.295600	-4.963400
H	-4.672400	-0.206600	-4.850000
H	-2.954800	-0.441500	-5.236100
C	-3.772200	1.441800	-5.952700
H	-4.051700	1.063600	-6.940300
H	-4.516500	2.179400	-5.641000
H	-2.797700	1.930100	-6.035200
O	-3.421200	0.788600	-3.643200
O	-1.196400	0.616300	-3.957500

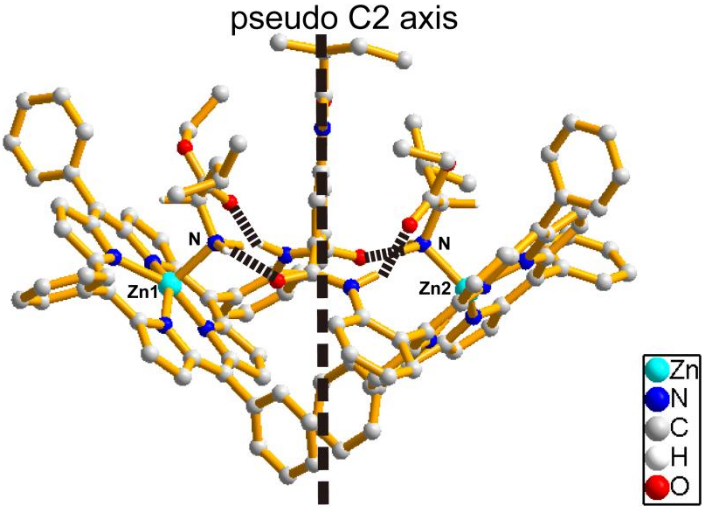

[Zn_2(S-MAABis)(L-LeuOEt)$_2$]

ΔE^{θ} = -9315.9466639 Hartree

Cartesian coordinates for Geometry Optimized [Zn_2(S-MAABis)(L-LeuOEt)$_2$]

Atoms	x	y	z
Zn	4.651500	-0.375400	-0.971800
N	3.348200	-0.857400	-2.442300
N	5.070500	1.332500	-1.973500
N	6.504900	-0.192100	-0.100700
N	4.857800	-2.400500	-0.641900
C	2.619300	-2.022900	-2.552800
C	2.723200	0.051600	-3.274400
C	4.261400	1.923200	-2.920700
C	6.057000	2.261100	-1.700600
C	7.273000	0.957400	-0.026200
C	7.092600	-1.077900	0.787000
C	5.682600	-3.009200	0.289500
C	3.958900	-3.380000	-1.022400
C	1.506200	-1.840800	-3.462300
H	0.784500	-2.589400	-3.733800
C	1.570400	-0.562400	-3.902900
H	0.922600	-0.071700	-4.602400
C	4.769400	3.235100	-3.271400
H	4.333500	3.885100	-4.009500
C	5.879700	3.440900	-2.523500

H	6.537300	4.292600	-2.526300
C	8.325200	0.799400	0.956300
H	9.048900	1.554500	1.209400
C	8.213600	-0.455700	1.457300
H	8.832400	-0.937600	2.194700
C	5.299200	-4.394600	0.479300
H	5.795000	-5.083500	1.141800
C	4.234400	-4.622200	-0.327200
H	3.679600	-5.535700	-0.458300
C	2.884900	-3.208800	-1.883100
C	3.122900	1.361700	-3.489100
C	7.072700	2.113900	-0.767800
C	6.699600	-2.391400	0.997400
C	1.883600	-4.313000	-2.007400
C	0.678300	-4.198100	-1.292300
C	-0.302800	-5.182200	-1.403800
H	-1.237200	-5.057300	-0.877300
C	-0.070000	-6.297400	-2.203400
H	-0.834600	-7.060100	-2.287400
C	1.131400	-6.432400	-2.895100
H	1.307700	-7.302100	-3.516000
C	2.100600	-5.435000	-2.801100
H	3.031200	-5.512400	-3.349700
C	2.274100	2.239100	-4.343500
C	1.669300	3.357200	-3.762800
H	1.826200	3.544100	-2.707300
C	0.857400	4.192100	-4.524900
H	0.383100	5.045000	-4.056700
C	0.634400	3.907400	-5.870600
H	-0.009700	4.548300	-6.460400
C	1.236600	2.794700	-6.454900
H	1.070900	2.575800	-7.503000
C	2.061100	1.967000	-5.697300
H	2.545900	1.109300	-6.148600
C	7.994700	3.265400	-0.543700
C	7.521600	4.434900	0.057300
H	6.479900	4.490800	0.349200
C	8.378200	5.510800	0.272200
H	8.003400	6.413600	0.739800
C	9.715500	5.426600	-0.109800
H	10.382300	6.263700	0.057900
C	10.191900	4.263600	-0.711300

H	11.229300	4.196300	-1.016500
C	9.335200	3.188100	-0.929800
H	9.695200	2.282400	-1.403000
C	7.407100	-3.172700	2.055400
C	7.074300	-2.968400	3.397300
H	6.286300	-2.264200	3.633700
C	7.725600	-3.687000	4.396400
H	7.463900	-3.525200	5.435500
C	8.711700	-4.613600	4.061900
H	9.218800	-5.172200	4.839400
C	8.395800	-4.099800	1.723600
H	8.650700	-4.248600	0.681200
C	9.044400	-4.819700	2.724700
H	9.811800	-5.537700	2.461300
Zn	-4.651200	0.450500	-0.933200
N	-3.386200	1.009700	-2.416400
N	-5.156100	-1.158200	-2.043800
N	-6.482600	0.271200	-0.030700
N	-4.782700	2.456900	-0.453900
C	-2.656500	2.176600	-2.505400
C	-2.790800	0.130800	-3.301300
C	-4.353100	-1.730000	-3.006500
C	-6.192600	-2.051100	-1.849100
C	-7.326700	-0.825000	-0.063200
C	-6.981100	1.081800	0.975400
C	-5.522700	3.000200	0.582300
C	-3.896500	3.449900	-0.825000
C	-1.580200	2.029800	-3.465300
H	-0.866800	2.788200	-3.731500
C	-1.661900	0.768500	-3.950100
H	-1.044700	0.305600	-4.695700
C	-4.904600	-3.000200	-3.435600
H	-4.474000	-3.631100	-4.193400
C	-6.047800	-3.190300	-2.734000
H	-6.743100	-4.008800	-2.801600
C	-8.340700	-0.712900	0.964800
H	-9.111000	-1.441200	1.150000
C	-8.120900	0.458500	1.612000
H	-8.681000	0.887500	2.425200
C	-5.104200	4.364600	0.845200
H	-5.545900	5.010900	1.585200
C	-4.101800	4.641400	-0.024200

H	-3.548000	5.558300	-0.134200
C	-2.878200	3.325800	-1.760200
C	-3.201700	-1.169600	-3.549600
C	-7.209900	-1.913700	-0.917100
C	-6.509300	2.345600	1.301000
O	-1.765500	-2.730600	-0.467800
O	1.827700	2.875000	-0.272500
C	-0.598100	-2.390600	-0.171500
C	0.660100	2.469100	-0.084000
N	3.355600	0.221700	0.463400
H	3.196100	1.233600	0.380100
H	2.457400	-0.243800	0.293800
C	3.884300	-0.210400	1.778800
H	4.973600	-0.224800	1.732100
C	3.404200	-1.626800	1.991200
C	3.451400	0.644000	2.988600
H	2.408800	0.428100	3.228000
H	4.064800	0.330500	3.840100
C	3.622900	2.156200	2.762100
H	2.947600	2.484900	1.962800
C	3.231800	2.893700	4.055800
H	3.291400	3.978000	3.919000
H	2.205400	2.641100	4.355500
H	3.906200	2.607900	4.871400
C	5.065200	2.497300	2.349900
H	5.200500	3.582300	2.295900
H	5.780700	2.093100	3.075200
H	5.311600	2.082900	1.367700
C	3.676300	-3.599200	3.367300
H	3.263200	-4.051600	2.466200
H	4.635100	-4.053200	3.615300
C	2.695000	-3.620100	4.529700
H	2.516600	-4.655300	4.837000
H	3.112500	-3.072800	5.380100
H	1.736000	-3.174700	4.255400
O	4.019500	-2.186500	3.053400
O	2.530200	-2.186200	1.332800
N	-3.302000	-0.268800	0.389900
H	-3.111200	-1.267300	0.227700
H	-2.424500	0.230800	0.211800
C	-3.766100	0.050000	1.763900
H	-4.854800	0.139000	1.746900

C	-3.208100	1.410900	2.095400
C	-3.391400	-0.962100	2.864700
H	-3.996600	-0.717200	3.743800
H	-2.341800	-0.847900	3.136200
C	-3.647600	-2.427400	2.469400
H	-2.930800	-2.718300	1.692300
C	-5.071500	-2.620000	1.920700
H	-5.273600	-3.684700	1.765700
H	-5.815100	-2.225200	2.623100
H	-5.211200	-2.112400	0.962100
C	-3.406200	-3.311000	3.707100
H	-2.409800	-3.130000	4.123200
H	-4.155300	-3.084000	4.475800
H	-3.493300	-4.370400	3.444300
C	-3.325000	3.253900	3.641800
H	-4.013000	3.477500	4.455200
H	-3.572500	3.850800	2.761500
C	-1.865000	3.435300	4.039700
H	-1.705200	4.462900	4.379700
H	-1.605900	2.751900	4.852700
H	-1.233600	3.244700	3.171400
O	-3.626000	1.836600	3.314000
O	-2.447200	2.066100	1.386600
N	0.533700	-3.063300	-0.467000
H	1.397600	-2.687200	-0.060400
N	-0.485300	3.099000	-0.427700
H	-1.350100	2.664200	-0.090700
C	-1.872500	4.429400	-1.861600
C	-0.650400	4.277800	-1.181300
C	0.322900	5.274000	-1.251500
H	1.266800	5.122700	-0.749500
C	0.069700	6.431600	-1.981600
H	0.829100	7.202100	-2.035600
C	-1.144500	6.599000	-2.643000
H	-1.336400	7.501500	-3.210100
C	-2.108500	5.593500	-2.585400
H	-3.051900	5.699300	-3.106400
C	-2.363400	-2.033200	-4.429000
C	-2.186900	-1.753900	-5.786300
H	-2.692500	-0.900300	-6.222400
C	-1.371200	-2.569400	-6.566400
H	-1.233200	-2.345500	-7.617500

C	-0.741500	-3.676100	-6.000000
H	-0.104300	-4.307600	-6.607300
C	-0.929500	-3.968400	-4.650700
H	-0.435900	-4.818700	-4.197800
C	-1.733400	-3.146500	-3.865700
H	-1.857500	-3.336200	-2.805800
C	-8.214100	-3.010900	-0.798100
C	-7.839500	-4.254400	-0.282700
H	-6.811900	-4.408300	0.023500
C	-8.776300	-5.277400	-0.166000
H	-8.478000	-6.238200	0.236200
C	-10.095600	-5.065200	-0.560600
H	-10.824800	-5.861100	-0.469000
C	-10.474300	-3.826700	-1.074600
H	-11.498000	-3.659000	-1.387300
C	-9.537500	-2.803800	-1.194700
H	-9.821400	-1.839100	-1.597900
C	-7.109100	3.034300	2.482400
C	-6.693900	2.674900	3.767500
H	-5.922500	1.922100	3.873700
C	-7.244400	3.302600	4.881800
H	-6.921800	3.017200	5.876500
C	-8.210900	4.293800	4.719800
H	-8.640400	4.781100	5.586900
C	-8.625700	4.654800	3.439600
H	-9.379400	5.422300	3.310200
C	-8.079100	4.025100	2.323600
H	-8.400100	4.292900	1.324100
C	0.017900	0.036100	-0.123900
C	-0.265800	-1.131500	0.580400
C	-0.231900	-1.177300	1.966800
C	0.350600	1.155300	1.991200
C	0.339300	1.176800	0.609200
H	-0.002100	0.056200	-1.209800
H	-0.422800	-2.095500	2.497100
H	0.592200	2.062100	2.533100
C	0.052500	-0.015700	2.686900
O	-0.333700	-2.165000	4.608100
N	0.065400	0.040300	4.089000
H	0.223100	0.958900	4.486700
C	-0.123100	-0.997600	4.970300
C	0.009700	-0.587600	6.428100

H	-0.313300	-1.461800	6.999100
C	1.496000	-0.294900	6.732200
H	1.854200	0.559100	6.146500
H	2.116800	-1.161900	6.489000
H	1.620500	-0.061200	7.793800
C	-0.888900	0.612700	6.791000
H	-0.468900	1.538500	6.373200
H	-0.853700	0.730800	7.879600
C	-2.347800	0.442900	6.329300
H	-2.439100	0.522500	5.242300
H	-2.981000	1.218400	6.771400
H	-2.735100	-0.534200	6.636600
